# Transcription factor E4F1 dictates spermatogonial stem cell fate decisions by regulating mitochondrial functions and cell cycle progression

**DOI:** 10.1186/s13578-023-01134-z

**Published:** 2023-09-25

**Authors:** Rong-Ge Yan, Zhen He, Fei-Chen Wang, Shuang Li, Qin-Bang Shang, Qi-En Yang

**Affiliations:** 1 Key Laboratory of Adaptation and Evolution of Plateau Biota, Northwest Plateau Institute of Biology, Chinese Academy of Sciences, Xining, 810001 China; 2 University of Chinese Academy of Sciences, Beijing, 100049 China; 3 Qinghai Key Laboratory of Animal Ecological Genomics, Northwest Plateau Institute of Biology, Chinese Academy of Sciences, Xining, 810001 China

**Keywords:** Spermatogenesis, Spermatogonial stem cell, Oxidative phosphorylation, Metabolism, Fate decisions, Transcription factor

## Abstract

**Background:**

Spermatogonial stem cells (SSCs) provide a foundation for robust and continual spermatogenesis in mammals. SSCs self-renew to maintain a functional stem cell pool and differentiate to supply committed progenitors. Metabolism acts as a crucial determinant of stem cell fates; however, factors linking metabolic programs to SSC development and maintenance are poorly understood.

**Results:**

We analyzed the chromatin accessibility of undifferentiated spermatogonia at the single-cell level and identified 37 positive TF regulators that may have potential roles in dictating SSC fates. The transcription factor E4F1 is expressed in spermatogonia, and its conditional deletion in mouse germ cells results in progressive loss of the entire undifferentiated spermatogonial pool. Single-cell RNA-seq analysis of control and *E4f1*-deficient spermatogonia revealed that E4F1 acts as a key regulator of mitochondrial function. E4F1 binds to promotors of genes that encode components of the mitochondrial respiratory chain, including *Ndufs5, Cox7a2, Cox6c,* and *Dnajc19.* Loss of *E4f1* function caused abnormal mitochondrial morphology and defects in fatty acid metabolism; as a result, undifferentiated spermatogonia were gradually lost due to cell cycle arrest and elevated apoptosis. Deletion of *p53* in *E4f1*-deficient germ cells only temporarily prevented spermatogonial loss but did not rescue the defects in SSC maintenance.

**Conclusions:**

Emerging evidence indicates that metabolic signals dictate stem cell fate decisions. In this study, we identified a list of transcription regulators that have potential roles in the fate transitions of undifferentiated spermatogonia in mice. Functional experiments demonstrated that the E4F1-mediated transcription program is a crucial regulator of metabolism and SSC fate decisions in mammals.

**Graphical Abstract:**

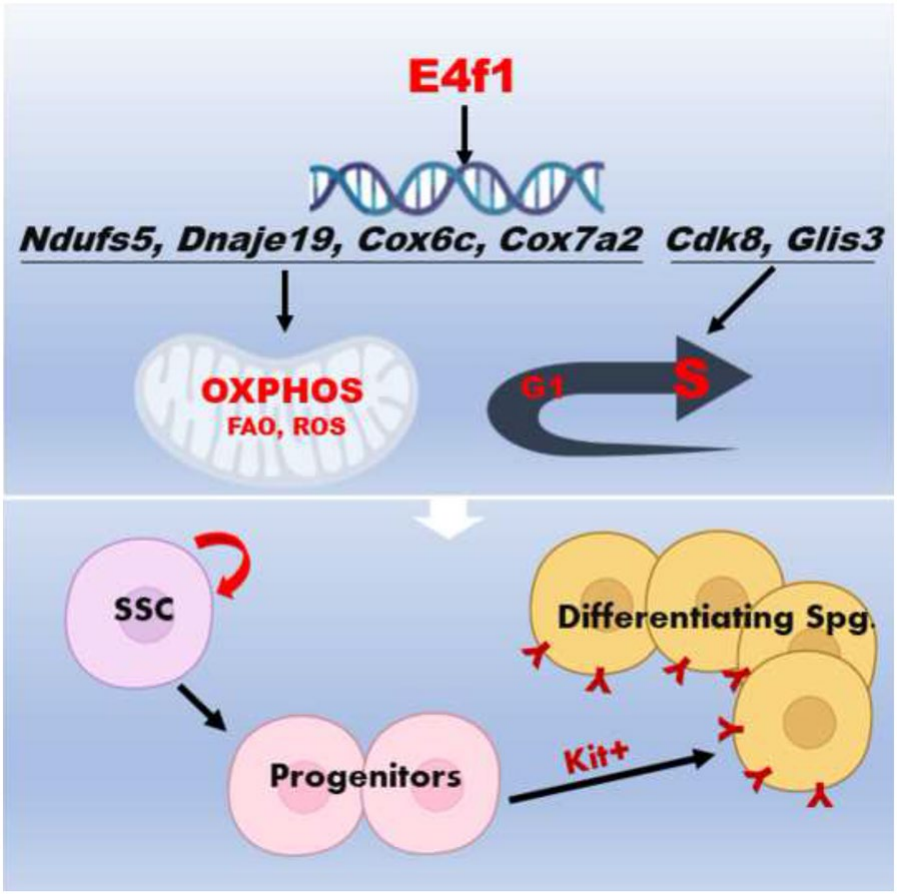

**Supplementary Information:**

The online version contains supplementary material available at 10.1186/s13578-023-01134-z.

## Background

Spermatogenesis is a classic stem cell-dependent system that generates millions of sperm daily throughout the life of the male. The lifelong maintenance of male fertility relies on functional spermatogonial stem cells (SSCs), which reside in undifferentiated spermatogonia and possess the ability to self-renew and differentiate to produce committed progenitors [1, 2]. Undifferentiated spermatogonia are a heterogeneous population consisting of A_single_ (A_s_), A_paired_ (A_pr_) and A_aligned_ (A_al_) spermatogonia. A small fraction of these cells contains enriched SSC activity, and the rest are committed progenitors [3]. SSC fate choice is a complicated process, and different models have been proposed [4, 5]; however, how distinct fates within closely related undifferentiated spermatogonial subpopulations are determined remains to be explored.

Metabolic signals are key regulators of stem cell fate decisions. Pluripotent stem cells show high glycolytic activity and low levels of oxidative phosphorylation (OXPHOS) under aerobic conditions [6, 7]. Slow cycling hematopoietic stem cells (HSCs) depend on glycolysis to maintain the quiescent state and switch to OXPHOS upon the initiation of differentiation [8]. Transcriptome analysis provides evidence that genes regulating glycolysis are elevated in the SSC-containing cell fraction [9], and in cultures, conditions favoring glycolysis enhance the long-term maintenance of SSC activities [10]. Functional experiments indicated that glycolysis plays key roles in SSC self-renewal and long-term maintenance. The forkhead box O (FOXO) transcription factor FOXO1, which regulates metabolism and mitochondrial respiration [11], is exclusively expressed in undifferentiated spermatogonia, and its deletion causes SSC loss in the postnatal mouse testis [12]. FOXO1 controls the expression of Myc/Mycn to maintain glycolytic activity and promote the self-renewal division of SSCs [13]. In contrast, progenitor spermatogonia rely on mitochondrial OXPHOS to complete successful fate transition into a differentiating state [14]. Disrupting mitochondrial fusion and inhibiting the metabolic switch impairs spermatogonial differentiation [15, 16]. Despite these findings, further studies are warranted to decipher the mechanisms that determine metabolic requirements in SSCs and progenitor spermatogonia.

E4 transcription factor 1 (E4F1) is a multifunctional protein that plays novel roles in cell cycle control, metabolism, and cell fate decisions. E4F1 was originally identified as a target of the E1A viral oncoprotein [17], and it was then discovered to be essential for cell cycle progression in stem cells [18]. E4F1 interacts with the cell cycle regulators pRB, p53 and checkpoint kinase 1 (CHK1) to perform its predominant roles in cell proliferation and survival [19, 20]. E4F1 also physically and genetically associates with the chromatin remodeling factors Bmi1 and Brg1 [21–23]. Recent studies have revealed that E4f1 is a master regulator of cell metabolism and fate determination. For example, loss of *E4f1* function impacts the expression of genes involved in pyruvate oxidation and mitochondrial homeostasis [24]. Gene expression and metabolism analysis of *E4f1* knockout keratinocytes revealed that E4F1 directly controls the transcription of dihydrolipamide (*Dlat*), a subunit of the mitochondrial pyruvate dehydrogenase (PDH) complex [25]. Although E4F1 seems ubiquitously expressed, its function is cell type and context dependent. *E4f1* knockout leads to the exhaustion of the epidermal stem cell pool in skin [22]; however, its deficiency does not affect the stem cell compartment but instead causes the depletion of multipotent progenitors in the hematopoietic system [20]. Conditional deletion of *E4f1* in Sertoli cells did not cause severe defects in spermatogenesis, indicating its intriguing roles in different tissues [26]. Because of its essential roles in metabolism, deciphering the function of E4F1 in spermatogenic cells will facilitate the understanding of the metabolic control of SSC fate decisions.

Here, we present evidence that germline-specific *E4f1* depletion results in the progressive loss of the undifferentiated spermatogonial population and defects in spermatogonial differentiation. *E4f1* inactivation disrupted mitochondrial morphology and function, thereby disrupting ROS homeostasis, fatty acid metabolism, and OXPHOS in SSCs. These findings reveal a central role of the E4F1-directed metabolism program in germline stem cell maintenance and development of the spermatogenic lineage.

## Results

### Inactivation of* E4f1* in spermatogonia caused defects in spermatogenesis

**Fig. 1:**
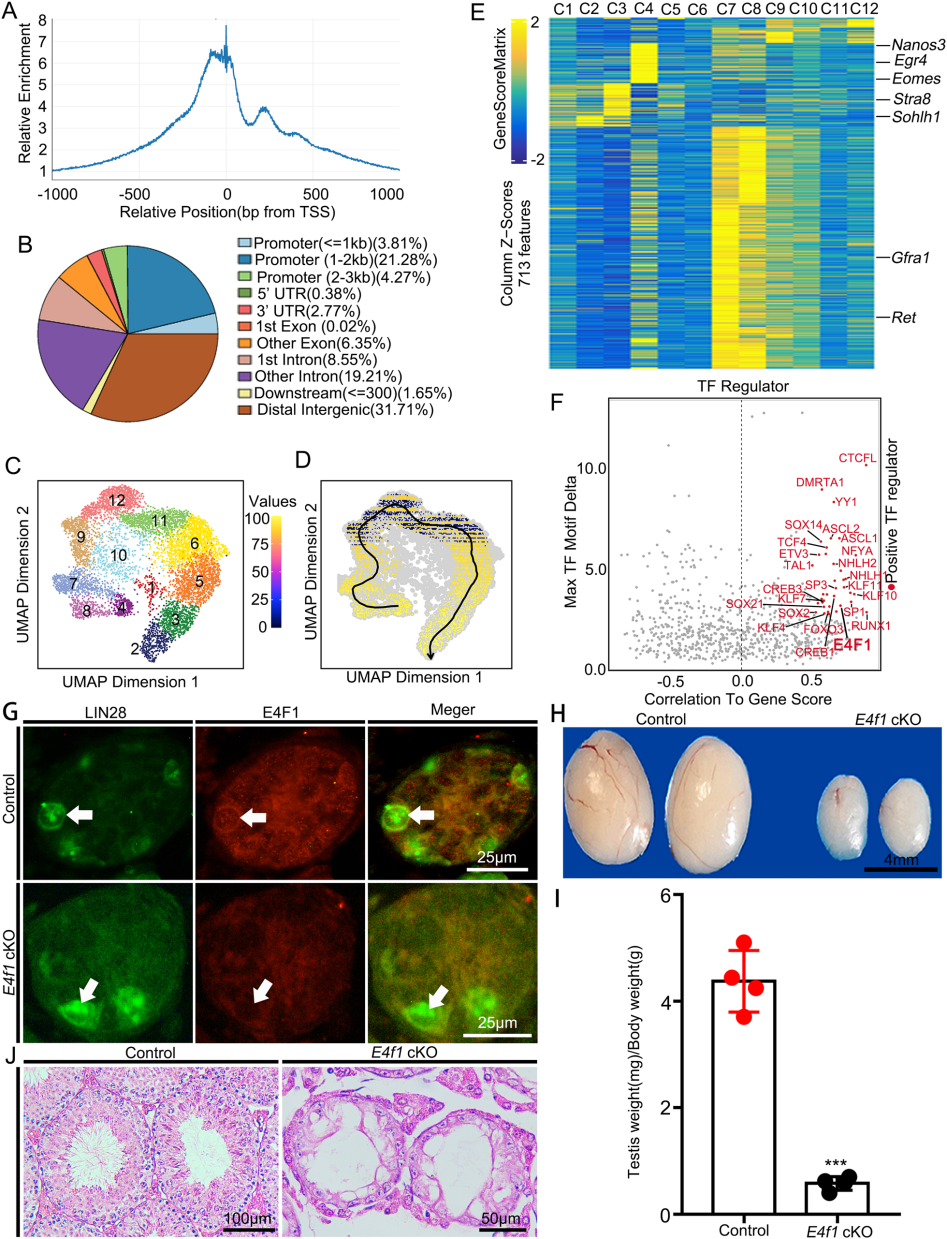
Inactivation of E4f1 in spermatogonia leads to abnormal spermatogenesis. **A** Peak distribution in the TSS ± 1 kb region. **B** Distribution of peaks in genomic functional areas. **C** UMAP visualization of the scATAC-seq dataset (7677 nuclei from Lin28-YFP-positive spermatogonia), colored by cluster. **D** Chromatin accessibility profiling of Lin28-YFP^+^ spermatogonia. The black line represents a double-spline fitted trajectory across chromatin accessibility profiling. **E** Heatmap showing dynamic gene scores across all clusters. **F** Plot of maximum difference between chromVAR deviation z score, depicting TF motif activity, against correlation of chromVAR deviation and corresponding TF score. TFs with maximum differences in chromVAR deviation z score in the top quartile of all TFs and a correlation of greater than 0.5 are indicated in red. **G** Immunofluorescence staining for E4F1 (red) and LIN28A (green) in cross-sections of control and *E4f1* cKO testes at postnatal day (PD) 6. **H** Gross morphology of testes from 2-month-old control and *E4f1* cKO mice. **I** The ratio of testis weight and body weight from 2-month-old controls and *E4f1* cKO mice. **J** H&E staining of control and *E4f1* cKO testes at 2 months of age. Error bars represent SD. **p < 0.01, ***p < 0.001, Student’s *t* test. n = 3

**Fig. 2:**
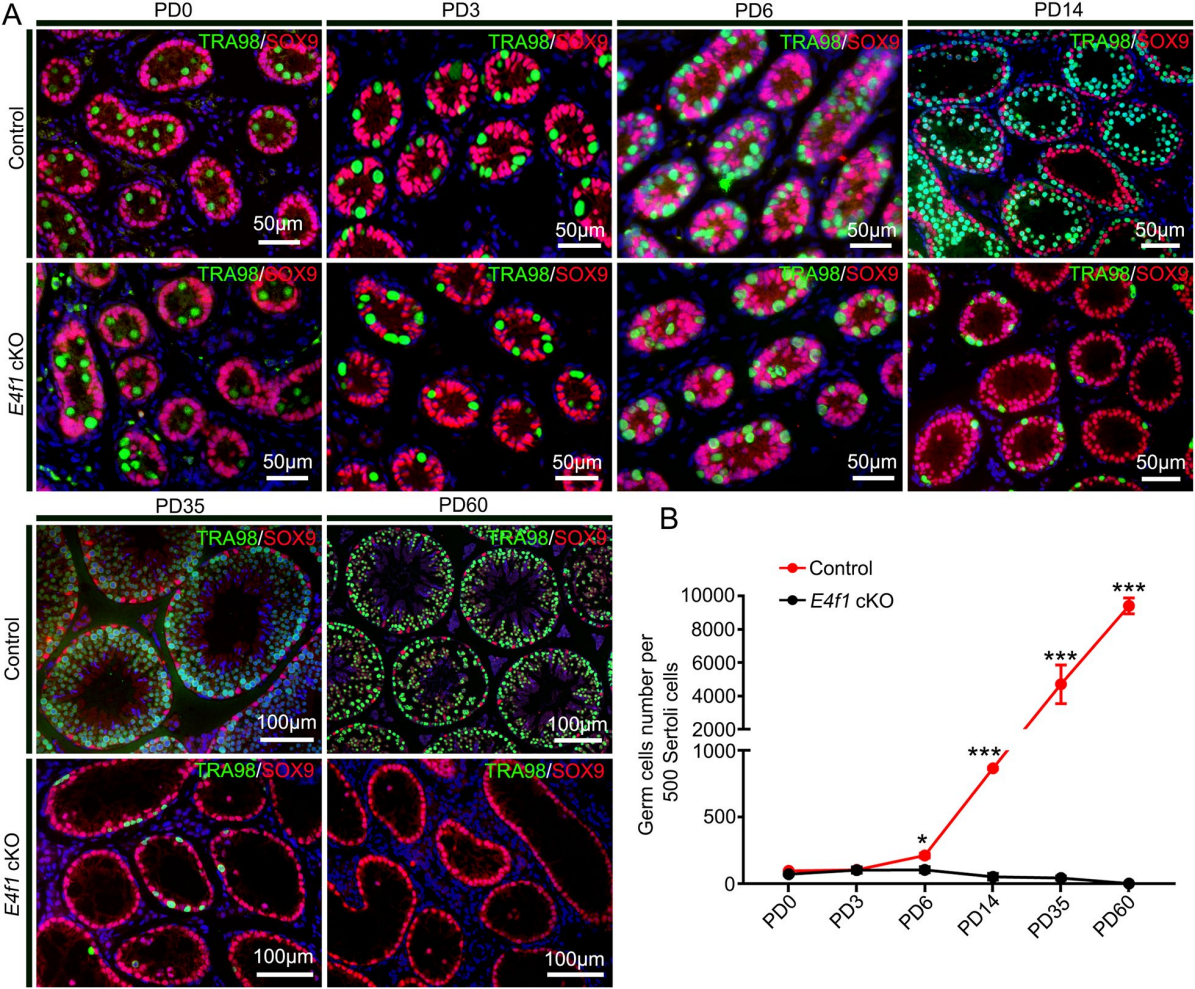
Inactivation of *E4f1* in spermatogonia leads to abnormal spermatogenesis. **A** Immunofluorescence staining for TRA98 (green) and SOX9 (red) in sections of testes from PD0 to adult controls and *E4f1* cKO mice. **B** Quantification of germ cells per 500 Sertoli cells in testis sections from PD0 to adult mice. Error bars represent SD. **p < 0.01, ***p < 0.001, Student’s *t* test. n = 3

To screen transcription regulators that have enriched motif accessibility in undifferentiated spermatogonia, we performed single-cell analysis of accessible chromatin (scATAC-seq) on isolated Lin28-YFP^+^ cells from 2-month-old mouse testes (n = 3). Lin28-YFP^+^ cells contained A_s_, A_pr_ and A_al_ undifferentiated spermatogonia and a proportion of differentiating spermatogonia [27]. A total of 7677 cells passed stringent quality control and were used for subsequent analysis. The median number of fragments per cell was 13704, 61.7% of fragments overlapped with targeted regions, and 51.5% of transposition events were in peaks. Detailed analysis of the peak distribution using ChIPseeker (v1.34.0) showed that 29.36% of ATAC peaks were enriched in the promoters of genes (Fig. 1A, B). We next generated a cluster visualization of cell subpopulations based on the detected peaks (Additional file 3: Table S1); as a result, 12 different clusters were identified (Fig. 1C). Among these clusters, C4 was the initial population, followed by C8 and C7, and cells from C2 were distributed at the end of the route (Fig. 1D). Predicted expression analysis (Genescore) based on peaks in the promoters of genes showed that reads in the promoters of undifferentiated spermatogonial-related genes (*Gfra1*, *Egr4*, *Nanos3*) were enriched in C4, C8 and C7, while peaks in the promoters of differentiating genes (*Stra8* and *Sohhlh1*) were observed in C2 (Fig. 1E & Additional file 4: Table S2). Integrated analysis of transcript levels (scRNA-seq on isolated Lin28-YFP^+^ cells) and motif accessibility resulted in the discovery of 37 positive TF regulators (*P* value < 0.01 and correlation to gene score > 0.5) in undifferentiated spermatogonia (Fig. 1F and Additional file 5: Table S3). We particularly focused on E4F1 because this transcription factor has a crucial role in controlling metabolism in adult stem cells. We next examined the cellular localization of E4F1 in the testes by immunohistochemistry and showed that E4F1 expression was low in gonocytes but was expressed in a proportion of spermatogonia after birth (Additional file 1: Fig. S1A, B). Therefore, we generated a conditional knockout mouse model to investigate the functional role of E4F1 in spermatogenesis. *E4f1* floxed mice (*E4f1*^*fl/fl*^) were crossed with *Vasa*-Cre transgenic mice that expressed Cre recombinase in gonocytes (prespermatogonia) beginning at embryonic day (ED) 15.5 [28]. Immunofluorescence staining showed that the resulting conditional knockout mice (*Vasa*-Cre; *E4f1*^*fl/fl*^, designated hereafter as *E4f1* cKO) lacked the E4F1 protein in Lin28-positive (Lin28^+^) spermatogonia at postnatal day 6 (PD6) (Fig. 1G), and *E4f1* mRNA was not expressed in *E4f1* cKO (Additional file 1: Fig. S1C), indicating successful deletion of *E4f1* in the germline. The testis weight of *E4f1 cKO* animals was significantly reduced, and the testis weight to body weight ratio was decreased by 86.73% at 2 months of age compared with that of littermate controls (*Vasa*-Cre; *E4f1*^*fl/*+^) (Fig. 1H, I). Histological analyses of seminiferous tubules revealed that spermatogenesis was intact in control mice, and in sharp contrast, spermatogenic cells were lost and spermatogenesis was severely disrupted in *E4f1 cKO* animals. It appeared that all seminiferous tubules examined at 2 months of age completely lacked germ cells, containing Sertoli cells only (Fig. 1J). To further determine the developmental stage at which germ cell loss occurred in *E4f1 cKO* mice, the number of TRA98^+^ germ cells per 500 SOX9^ +^ Sertoli cells was quantified at postnatal days (PDs) 0, 3, 6, 14, 35 and 60 of age (Fig. 2A). The results showed that the germ cell number was similar between control and *E4f1 cKO* mice at PD3, indicating that deletion of *E4f1* did not affect the development of prospermatogonia in prenatal and neonatal testes. A reduction in total germ cell number was first evident at PD6 and continued at PD35 until the complete elimination of all germ cells at PD60 (Fig. 2B and Additional file 1: Fig. S1D). The ratio of GFRA1^+^ spermatogonia in *E4f1 cKO* mice increased significantly (341.3 ± 39.44 vs 122.5 ± 14.79 control) after birth compared with that in control mice (Additional file 1: Fig. S1E&F), indicating that the development of progenitor spermatogonia was abnormal. Germ cell loss from PD3 to PD6 was not due to defects in cell migration because the number of germ cells that reached the basement membrane was similar between control and *E4f1 cKO* mice (Additional file 1: Fig. S1G, H). Together, these findings demonstrated that E4F1 was essential for the first round of spermatogenesis and sustained the entire spermatogenic lineage in mice.

### *E4f1* inactivation affected the fate decisions of undifferentiated spermatogonia

**Fig. 3:**
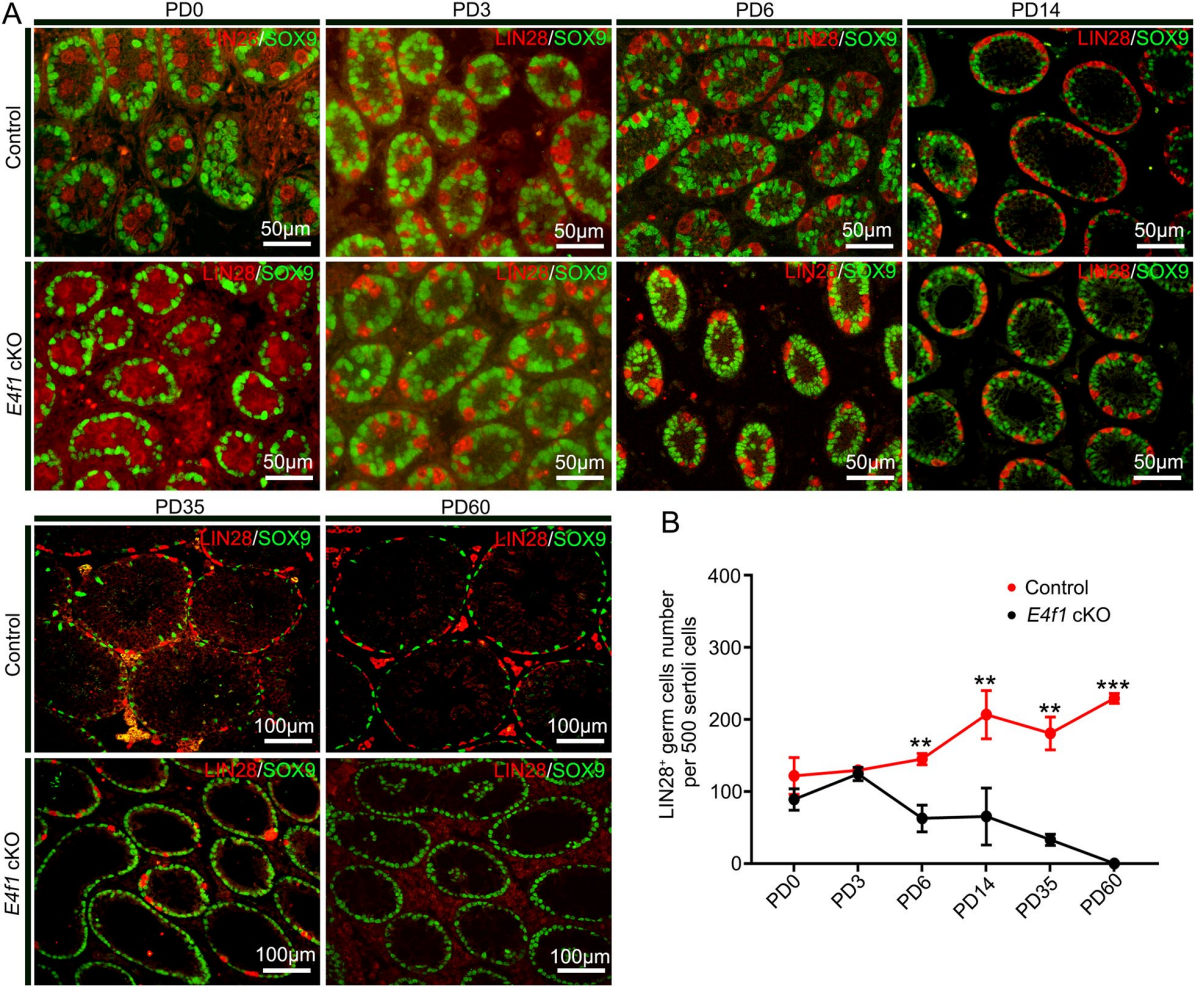
Genetic inactivation of *E4f1* blocked spermatogonia development. **A** Immunofluorescence staining for SOX9 (green) and LIN28A (red) in sections of testes from PD0 to adult controls and *E4f1* cKO testes. **B** Quantification of undifferentiated spermatogonia per 500 Sertoli cells in testis sections from PD0 to adult control and *E4f1* cKO mice. n = 3. Error bars represent SD. **P < 0.01, ***P < 0.001, Student’s *t* test

**Fig. 4:**
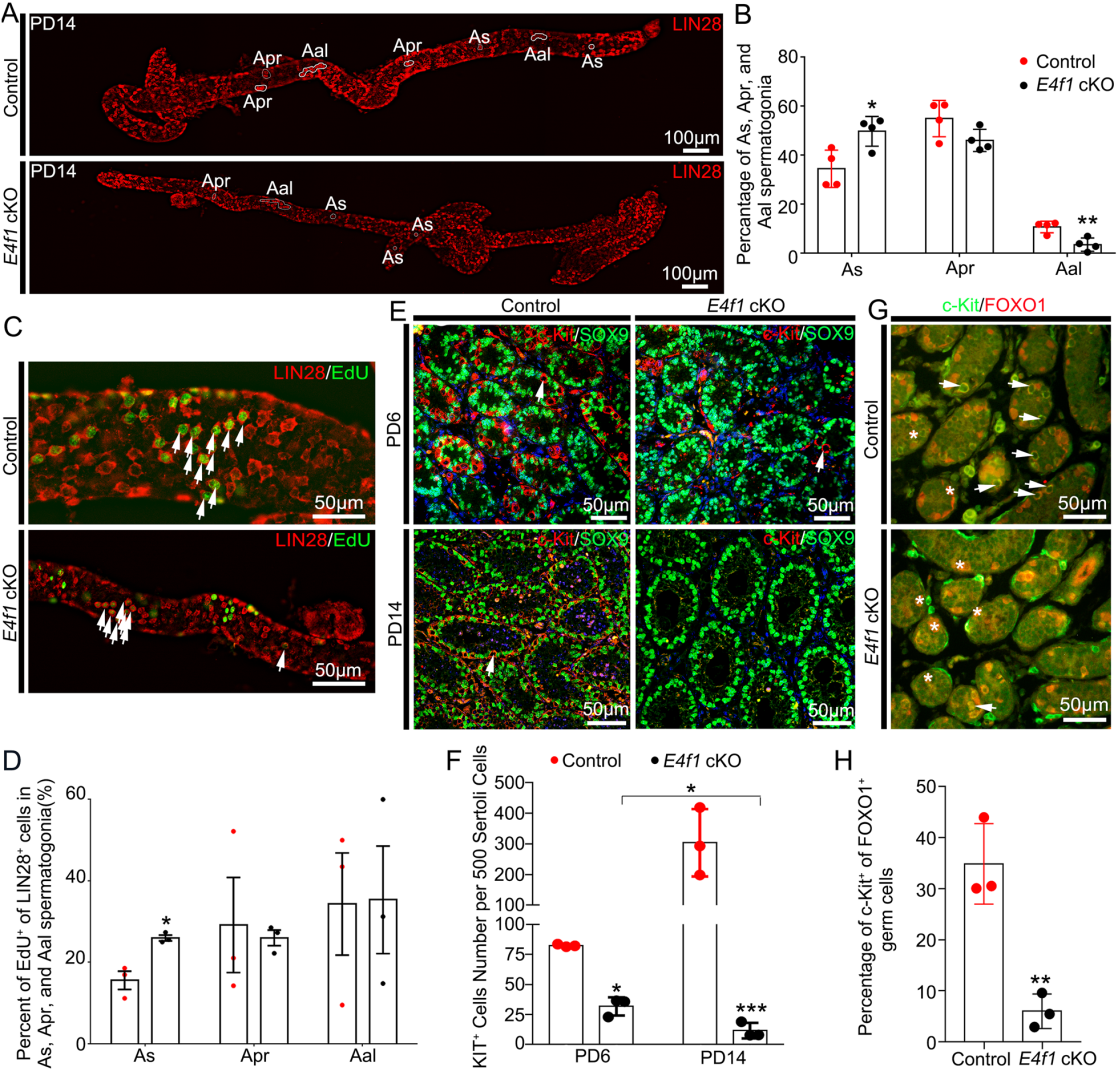
Genetic inactivation of *E4f1* blocked spermatogonia development. **A** Whole mounts of seminiferous tubules from PD14 control and *E4f1* cKO mice stained with LIN28. **B** The percentage of A_s_, A_pr_, and A_al_ spermatogonia. **C** Immunofluorescence staining for EdU (green) and LIN28 (red) in sections of PD14 control and *E4f1* cKO testes. **D** The percentage of EdU^+^ LIN28^+^ cells in A_s_, A_pr_, and A_al_ spermatogonia. **E** Immunofluorescence staining for c-Kit (red) and SOX9 (green) in sections of PD6 and PD14 control and *E4f1* cKO testes. **F** Quantification of c-Kit^+^ spermatogonia per 500 Sertoli cells in testis sections from PD6 and PD14 control and *E4f1* cKO mice. n = 3. **G** Immunofluorescence staining for KIT (green) and FOXO1 (red) in sections of PD6 control and *E4f1* cKO testes. **H** Quantification of Kit^+^ spermatogonia of FOXO1^+^ germ cells in testis sections from PD6 control and *E4f1* cKO mice. n = 3. Error bars represent SD. *P < 0.05, **P < 0.01, ***P < 0.001, Student’s *t* test

Considering that progressive loss of germ cells is usually due to defects in SSC fate decisions, we next examined whether undifferentiated spermatogonia, which contain SSCs and progenitors, were affected by *E4f1* knockout. We quantified the number of undifferentiated spermatogonia (LIN28^+^ cells) in testis cross-sections and found that the number of LIN28^+^ cells per 500 Sertoli cells for *E4f1* cKO mice was only 43.2% of that for control mice (62.7 ± 10.7 vs 145.0 ± 4.5 control) at PD6, indicating a decline in the size of the undifferentiated spermatogonial pool. This number further declined to 31.6% at PD14 (65.3 ± 22.8 vs 206.7 ± 19.4 control) (Fig. 3A, B). The undifferentiated spermatogonia population consists of different subtypes of cells, including A_s_, A_pr_ and A_al_ spermatogonia. We next examined the percentage of each subset using whole-mount immunofluorescence staining. The percentage of A_s_ spermatogonia was increased (49.7 ± 3.0 vs 34.4 ± 3.8 control), but the percentage of A_al_ spermatogonia was decreased (3.4 ± 1.4 vs 10.7 ± 1.2 control) in control and *E4f1* cKO testes at PD14 (Fig. 4A, B). The percentage of proliferating A_s_ spermatogonial cells also increased (25.98 ± 0.71 vs 15.57 ± 2.2 control) in *E4f1* cKO testes at PD14 compared with the control (Fig. 4C, D). Because the entire undifferentiated spermatogonial pool, including A_s,_ disappeared at 2 months of age, we speculated that a transient enrichment of A_s_ cells indicated that the balance between self-renewal and differentiation of SSCs was disrupted.

At stage VII of the spermatogenic cycle, a proportion of undifferentiated spermatogonia enter the differentiation pathway and become c-Kit^+^ differentiating spermatogonia [29]. We next examined spermatogonial differentiation and found that the number of c-Kit^+^ germ cells within the seminiferous tubules of *E4f1* cKO mice was significantly decreased. In control testes, the number of c-Kit^+^ spermatogonia per 500 Sertoli cells was 82.5 ± 0.6 at PD6 and 303.5 ± 63.7 at PD14; however, this number was decreased to 31.8 ± 4.4 and 11.7 ± 3.7 in *E4f1* cKO testes, respectively (Fig. 4E, F). Meanwhile, the percentage of c-Kit^+^ in FOXO1^+^ germ cells decreased significantly in the testes of *E4f1* cKO mice at PD6 (6.02 ± 1.95 vs 34.84 ± 4.55 control, P < 0.05) (Fig. 4G, H). These findings indicated that the fate decisions of *E4f1*-deficient undifferentiated spermatogonia were impaired and that these cells lost the differentiation potency to generate differentiating spermatogonia. It is also possible that the survival of newly formed differentiating spermatogonia relies on E4F1 function.

### *E4f1* inactivation induced cell cycle arrest at the G1/S phase and apoptosis

**Fig. 5:**
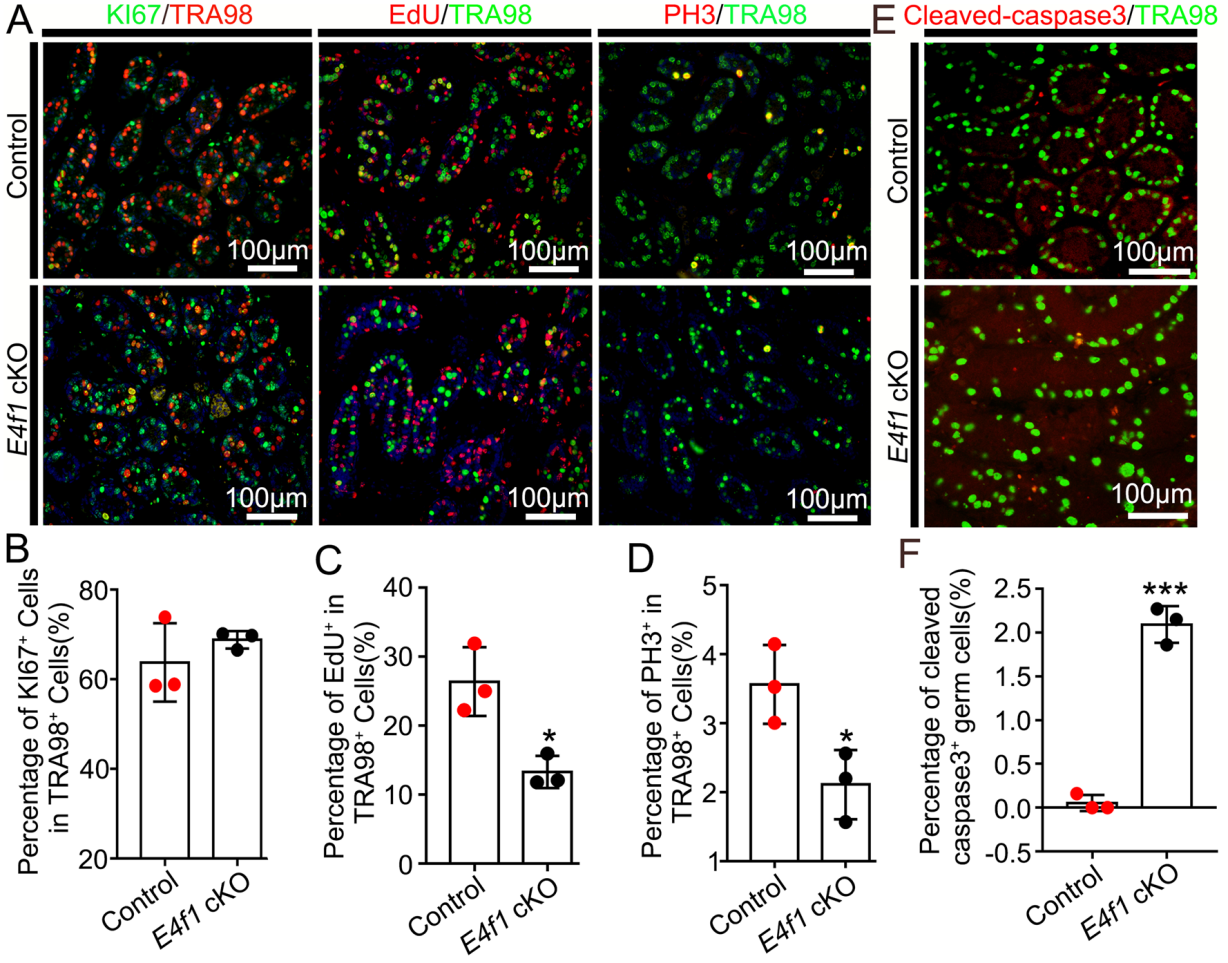
*E4f1* inactivation induced spermatogonia cell cycle blockade at G1/S phase and apoptosis **A** Immunofluorescence staining for KI67 (green) with TRA98 (red), EdU (red) with TRA98 (green), and PH3 (p-Histone H3, red) with TRA98 (green) in sections of PD6 controls and *E4f1* cKO testes. **B** Percentage of KI67^+^ germ cells in testis sections from PD6 controls and *E4f1* cKO mice. n = 3. **C** Percentage of EdU-positive germ cells in testis sections from PD6 controls and *E4f1* cKO mice. n = 3. (**D**) Percentage of PH3-positive germ cells in testis sections from PD6 controls and *E4f1* cKO mice. n = 3. **E** Immunofluorescence staining for cleaved caspase-3 (red) and TRA98 (green) in sections of PD6 control and *E4f1* cKO testes. **F** Percentage of apoptotic germ cells in testis sections from PD6 controls and *E4f1* cKO mice. n = 3. Error bars represent SD. *p < 0.05, ***p < 0.001, Student’s *t* test

Cell cycle regulation is a key part of stem cell fate decisions, and we wondered whether *E4f1* deletion impacted cell cycle progression in undifferentiated spermatogonia. First, considering that Ki67 is a marker of cells in the mitotic cell cycle except G0, double immunohistochemistry was performed to compare fractions of proliferative germ cells in cross-sections of testes from control and *E4f1 cKO* mice at PD6. The results showed that the fraction of Ki67^+^ germ cells did not differ between control and *E4f1 cKO* testes, suggesting that E4F1 was not involved in the G0 to G1 transition (Fig. 5A, B). Next, the percentages of germ cells in the S and M phases of the mitotic cell cycle were examined using EdU labeling and phospho-histone 3 (PH3) staining. The results showed that *E4f1* deletion greatly impacted the G1-S transition because EdU^+^ germ cells were reduced by 49.6% in *E4f1* cKO testes (13.3 ± 1.3 vs 26.4 ± 2.9 control) (Fig. 5A, C). Similarly, PH3^+^ germ cells in *E4f1* cKO testes were only 42.3% of those in controls (1.1 ± 0.3 vs 2.6 ± 0.3 control) (Fig. 5A and D).

Next, we isolated THY1^+^ testicular cells, which contain enriched SSCs [30], to examine the cell cycle dynamics using PI staining and flow cytometry (FACS) analysis. The cells from control testes contained a G0/G1 phase fraction of 64.42%, an S-phase fraction of 22.99%, and a G2-phase fraction of 12.59%. The fractions of G0/G1, S and G2 phases were changed to 83.92%, 13.48% and 2.60% for the cells from *E4f1* cKO testes (*P* < 0.05, Additional file 1: Fig. S2A&B). These data further demonstrated that *E4f1* deletion caused abnormal cell cycle progression, particularly arrested cells at G1/S phase. Meanwhile, the THY1^ +^ testicular cells showed elevated apoptosis upon *E4f1* deletion, as revealed by Annexin V staining (61.8 ± 0.7 vs 50.4 ± 1.5 control) (Additional file 1: Fig. S2C&D), which was consistent with the immunofluorescence staining (0.053 ± 0.053 vs 2.093 ± 0.122 control) (Fig. 5E, F). Collectively, these findings suggest that *E4f1* inactivation not only compromises SSC maintenance but also blocks progenitor spermatogonia expansion, spermatogonial differentiation and survival.

### scRNA-seq provides insight into the transcriptomic changes in *E4f1*-deficient spermatogonia

**Fig. 6:**
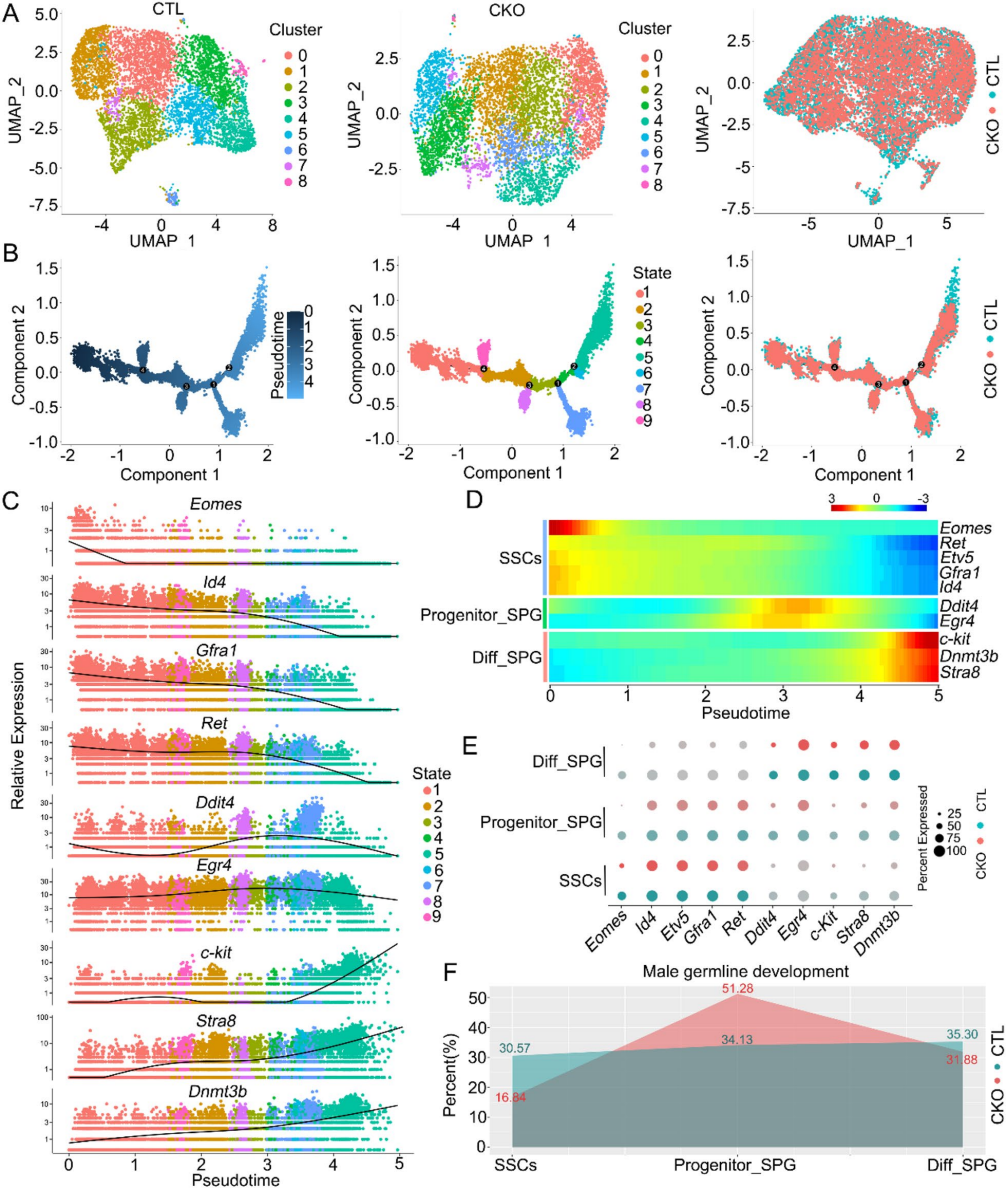
scRNA-seq defines the transcriptomes of *E4f1* cKO spermatogonia. **A** The resulting UMAP plots show unbiased clustering of germ cells extracted from testicular cells of controls and *E4f1* cKO mice and the resulting UMAP plots of integrated germ cells of control and *E4f1* cKO testes. **B** Integrated germ cell trajectories with cells ordered in pseudotime, states, and origin by unbiased dynamic lineage analysis. **C** Expression levels (vertical axis) of marker genes during spermatogonia development among integrated germ cells ordered in pseudotime (cell coloring id according to states from (B)). **D** Heatmap of marker gene expression patterns corresponding to the pseudotime trajectory of integrated germ cells. **E** Dotplot for the expression of selected marker genes across germ cell types in controls and *E4f1* cKO mice. **F** Summary schematic depicting the percentage of spermatogonia in each cellular state in controls and *E4f1* cKO mice

Phenotypic analysis provided compelling evidence that E4F1 is key for SSC maintenance and lineage decisions. We next examined the patterns and dynamics of gene expression within different undifferentiated spermatogonial subtypes in the testes of *E4f1* cKO mice. Single cells of testes from 3 control and 3 *E4f1* cKO mice were isolated and processed for scRNA-seq analysis using the BD Rhapsody™ Single-Cell Analysis System [31]. Single-cell analysis was performed on PD6 testes, which contain enriched spermatogonia [32]. A total of 20280 control and 15526 *E4f1* cKO cells passed standard quality control and were retained for subsequent analysis (Additional file 1: Fig. S3). On average, 46322.9 and 34835.5 reads, 7793.14 and 7657.845 unique molecular indices (UMIs), and 22329 and 21403 mean genes were detected per library for control and *E4f1* cKO testes, respectively (Additional file 1: Fig. S3C). Spermatogonial cells were extracted by the expression of germ cell markers (*Ddx4* and *Dazl*) for clustering and differential gene expression [33] analysis (Additional file 1: Fig. S3). A total of 6323 and 6226 germ cells were obtained from control and *E4f1* cKO mice, respectively. Uniform Manifold Approximation and Projection (UMAP) analysis identified 9 clusters for control and 9 clusters for *E4f1* cKO germ cells (Fig. 6A). Data were integrated to uncover the difference in gene expression at single-cell resolution between control and *E4f1* cKO germ cells, and as a result, 11 different clusters were identified. Unbiased dynamic cell trajectory analysis of pseudotime order with integrated cells yielded developmental continua with a major branching point and 9 distinct states (Fig. 6B, Additional file 6: Table S4). Pseudotime profiles were scrutinized based on genes that distinguish undifferentiated spermatogonia (*Eomes*, *Id4*, *Etv5*, *Gfra1*, *Ret*, *Ddit4*, *Egr4*) from spermatogonia that enter the differentiation pathway (*Kit*, *Stra8*, *Dnmt3b*) (Fig. 6C, D). The expression of genes specific to SSCs and differentiating spermatogonia was skewed preferentially toward the beginning (state 1) and end of the trajectories (states 4, 5, 6 and 7). These cells were further scrutinized, and the results showed that cells in state 1 expressed elevated levels of genes associated with SSCs and that those in states 2, 3, 8, and 9 were likely progenitor spermatogonia. Cells in States 4, 5, 6 and 7 were differentiating spermatogonia (Fig. 6C, E). Although the total number of spermatogonia was decreased, we detected that the ratios of SSCs and progenitors were increased and the ratio of differentiating spermatogonia was decreased (Fig. 6F), consistent with the observation that A_s_ spermatogonia were enriched in *E4f1* cKO testes at PD6.

**Fig. 7:**
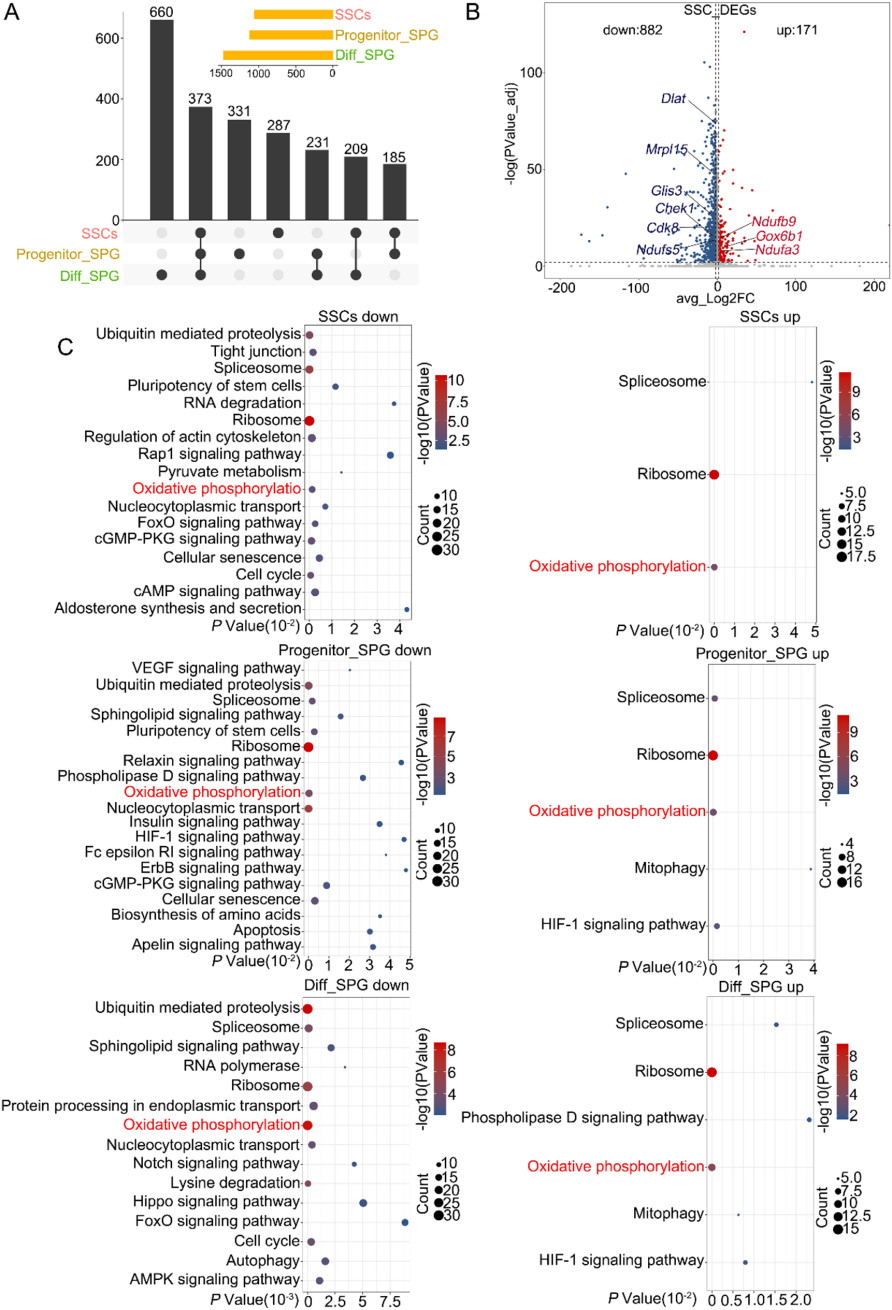
E4f1 controls a limited set of genes involved in mitochondrial homeostasis, the cell cycle and survival. **A** Number of DEGs between control and *E4f1* cKO germ cells in different cell types. Black circles represent DEGs in corresponding cell types. **B** Volcano map showing differentially expressed genes in SSCs between controls and *E4f1* cKO mice. **C** Enriched KEGG pathways in the downregulated genes (left) and upregulated genes [60] between control and *E4f1* cKO spermatogonia

After clarifying the identity of cell fates, we next analyzed DEGs in the SSC-containing population, progenitor spermatogonia and differentiating spermatogonia between control and *E4f1* cKO mice. The results showed that the expression of 1054, 1120, and 1473 genes were altered by *E4f1* deletion in these three distinct spermatogonial populations (Fig. 7A, Additional file 7: Table S5), indicating that a significantly higher number of genes was dependent on *E4f1* in the SSC-enriched spermatogonial population. The volcano map showed that 882 genes were downregulated and 171 were upregulated in *E4f1*-null SSCs (Fig. 7B). The expression of genes involved in SSC maintenance, including *Glis3*, *Huwe1*, *Ehmt2*, and *Phf13,* was changed by *E4f1* deletion. KEGG pathway analysis identified different GO terms for these DEGs in SSCs, progenitor spermatogonia and differentiating spermatogonia (Fig. 7C, Additional file 8: Table S6). Oxidative phosphorylation was enriched in all three subtypes of undifferentiated spermatogonia, and notably, the relative abundances of *Ndufs5*, *Ndufv1*, *Ndufa13*, *Ndufa3*, *Cox7a2*, *Uqcr11*, *Uqcrq*, *Cox6c*, *Atp5g1*, and *Cox7a2l* transcripts were significantly different in control and *E4f1*-depleted spermatogonia. In addition, genes associated with the spliceosome pathway and ribosome were upregulated in three subsets of cells, indicating the potential role of E4f1 in mRNA processing and translation. Taken together, single-cell transcriptional analysis of male germ lineages indicates that *E4f1* deficiency affects the molecular identities and gene expression dynamics of SSCs and their progeny.

### *E4f1* deletion caused mitochondrial defects and changed metabolism in undifferentiated spermatogonia

**Fig. 8:**
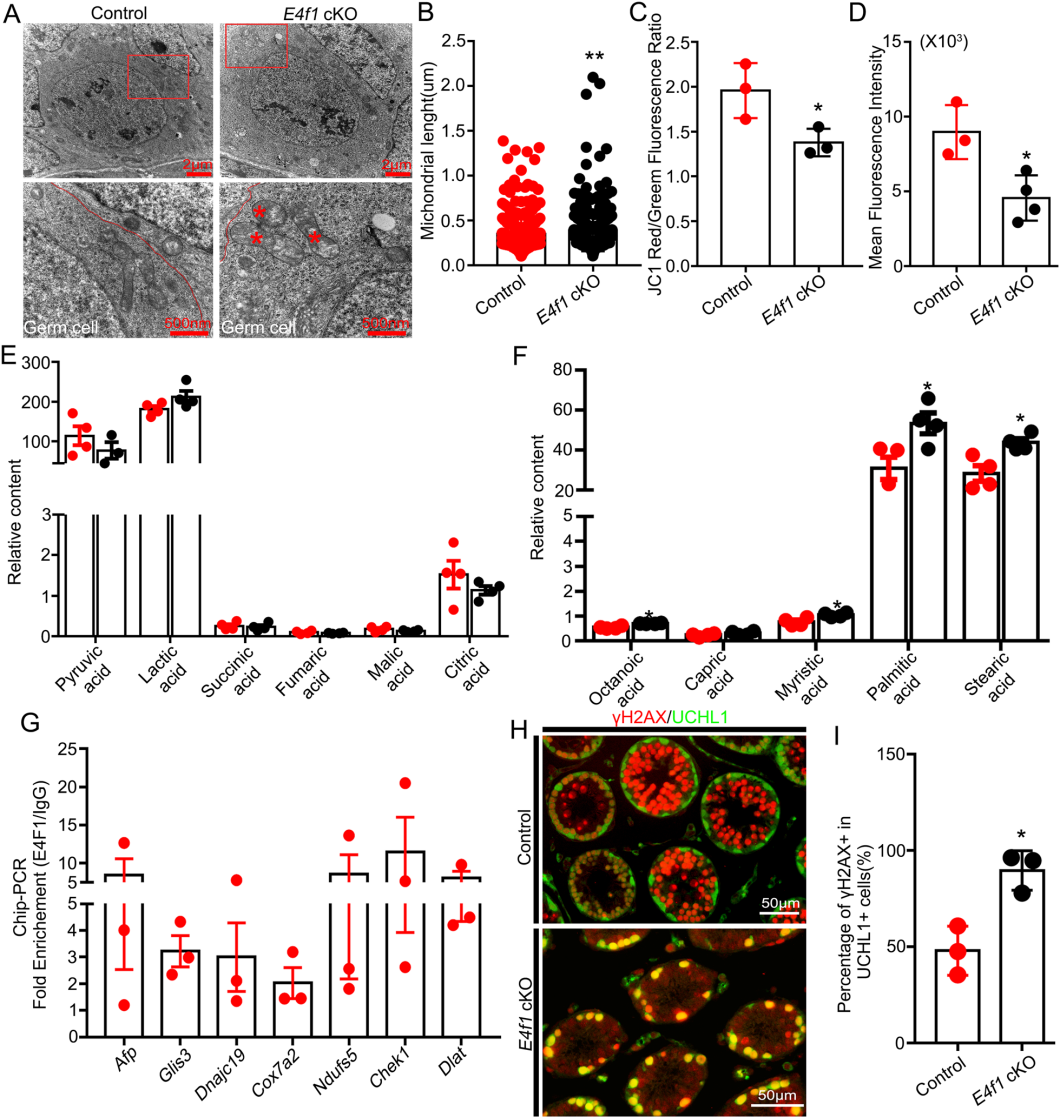
E4f1 regulated spermatogonia mitochondrial function and fatty acid metabolism. **A** Transmission electron microscopy images of spermatogonia from PD6 control and *E4f1* cKO testes. **B** Quantification of mitochondrial length in spermatogonia from PD6 control and *E4f1* cKO testes. **C** Quantification by FACS of the mean JC1 red/green fluorescence ratio values of THY1^+^ cells from PD6 control and *E4f1* cKO testes. **D** Quantification by FACS of the mean fluorescence intensity of MitoSOX™ red in THY1^+^ cells from PD6 control and *E4f1* cKO testes. **E** Relative intensity of metabolites involved with pyruvic acid in THY1^+^ cells from PD6-8 control and *E4f1* cKO testes. **F** Relative intensity of several fatty acids in THY1^+^ cells from PD6-8 control and *E4f1* cKO testes. **G** Fold enrichment of DNA obtained with ChIP analysis with E4F1 versus normal mouse IgG by real-time PCR. n >  = 3. Error bars represent SEM. *p < 0.05, Student’s *t* test. **H** Immunofluorescence staining for gH2AX (red) and UCHL1 (green) in sections of PD14 control and *E4f1* cKO testes. **I** Percentage of gH2AX-positive undifferentiated spermatogonia in testis sections from PD14 control and *E4f1* cKO testes. n = 3

We next aimed to determine whether mitochondrial function and OSPHOS were indeed impaired by *E4f1* deletion. Transmission electron microscopy (TEM) evaluation of spermatogonia from the testes of PD6 mice revealed defects in the mitochondrial morphology of *E4f1* cKO spermatogonia (Fig. 8A). Mitochondria in the spermatogonia of control animals were intact, while those in *E4f1* cKO mice were swollen and increased in length (Fig. 8A&B). Mitochondrial membrane potential (MMP), which indicates electron transport and OXPHOS [34], was reduced by 29.6% in SSCs containing THY1^+^ testicular cells of *E4f1* cKO animals (1.38 ± 0.09 vs 1.96 ± 0.18 control, n = 3, *P* < 0.05) (Fig. 8C). We then measured mitochondrial ROS levels using MitoSox staining [35] and found that ROS production was reduced by 48.1% in *E4f1*-depleted spermatogonia (4559 ± 757.9 vs 8976 ± 1043 control, n = 3, *P* < 0.05) (Fig. 8D and Additional file 1: Fig. S4A). Because ROS is required for spermatogonial stem cell maintenance and supplementation with physiological levels (30 µM) of H_2_O_2_ increased spermatogonial proliferation [36], we next treated cultured control and *E4f1* cKO testes with H_2_O_2_ but did not observe significant effects (Additional file 1: Fig. S4B&4C). Collectively, these data demonstrated that mitochondrial integrity and activity were impaired by *E4f1* deletion.

E4F1 regulates pyruvate dehydrogenase activity to impact mitochondrial function in skin cells [25]. We next measured the concentrations of key metabolites in THY1 ^+^ cells using Gas Chromatography Mass Spectrometry (GC–MS). The metabolites related to pyruvate metabolism included pyruvic acid, lactic acid, succinic acid, fumaric acid, malic acid, and citric acid. The results revealed that lactate (212.4 ± 14.81 vs 181.3 ± 8.06 control, n = 4, *P* = 0.1147) and pyruvate acid concentrations (76.87 ± 20.94 vs 113.8 ± 24.02 control, n = 4, *P* = 0.3192) did not change in *E4f1* cKO cells compared to control cells (*P* > 0.05) (Fig. 8E); however, octanoic acid, capric acid, myristic acid, palmitic acid, and stearic acid were increased in *E4f1* cKO cells (Fig. 8F), indicating that defects in mitochondrial activities caused the accumulation of fatty acids [37].

To gain insight into the E4F1-dependent gene expression programs that control metabolism and cell cycle progression in spermatogonia, we screened DEGs of the scRNA-seq dataset that contain the E4F1 motif in the promoters [38] and identified 174 candidate genes (Additional file 9: Table S7), including genes that play important roles in mitochondrial function (*Dlat*, *Ndufs5*, *Mrpl15*, *Cox7a2*, *Dnajc9*, *Dnajc19*, *Mrpl48*, *Cox6c*, *Mpc2*, *Atp2b1*), the cell cycle (*Ccng1*, *Cdk8*, *Chek1*) and fatty acid binding (*Afp*). *Glis3*, a transcription factor that is exclusively expressed in undifferentiated spermatogonia and plays an essential role in sustaining the SSC population [39], was predicted to be an E4F1 target. The relative abundances of these transcripts, except *Afp,* were downregulated by *E4f1* deletion in spermatogonial cells, further confirming that E4f1 directly participates in multiple cellular events involving mitochondrial function, metabolism, and the cell cycle. A set of these genes (*Afp*, *Dlat*, *Dnajc19*, *Cox7a2*, *Ndufs5*, *Chek1*, *and Glis3*) was further validated as direct targets for *E4f1* by ChIP‒qPCR experiments (Fig. 8G). Because *E4f1* deletion caused a decrease in ROS production, we next co-stained the spermatogonial marker UCHL1 with phospho-H2A. X (γH2A. X) to detect whether DNA damage occurred in the spermatogonial lineages [40, 41]. The results showed that *E4f1*-deficient spermatogonia still exhibited a strong immunoreactive signal for γH2A. X, which marks DNA damage and is normally enriched in leptotene and pachytene spermatocytes in response to meiotic DNA double-strand breaks in control testes (Fig. 8H, I). This is likely because E4F1 is directly recruited to DNA lesions to participate in DNA double-strand break repair, and its deletion causes DNA damage in spermatogonia [23]. These data indicated that *E4f1* directly binds to the promoters of a limited number of genes that play essential roles in maintaining mitochondrial function and cell cycle regulation.

### *Trp53 *deletion did not rescue the spermatogonial loss induced by *E4f1* inactivation

**Fig. 9:**
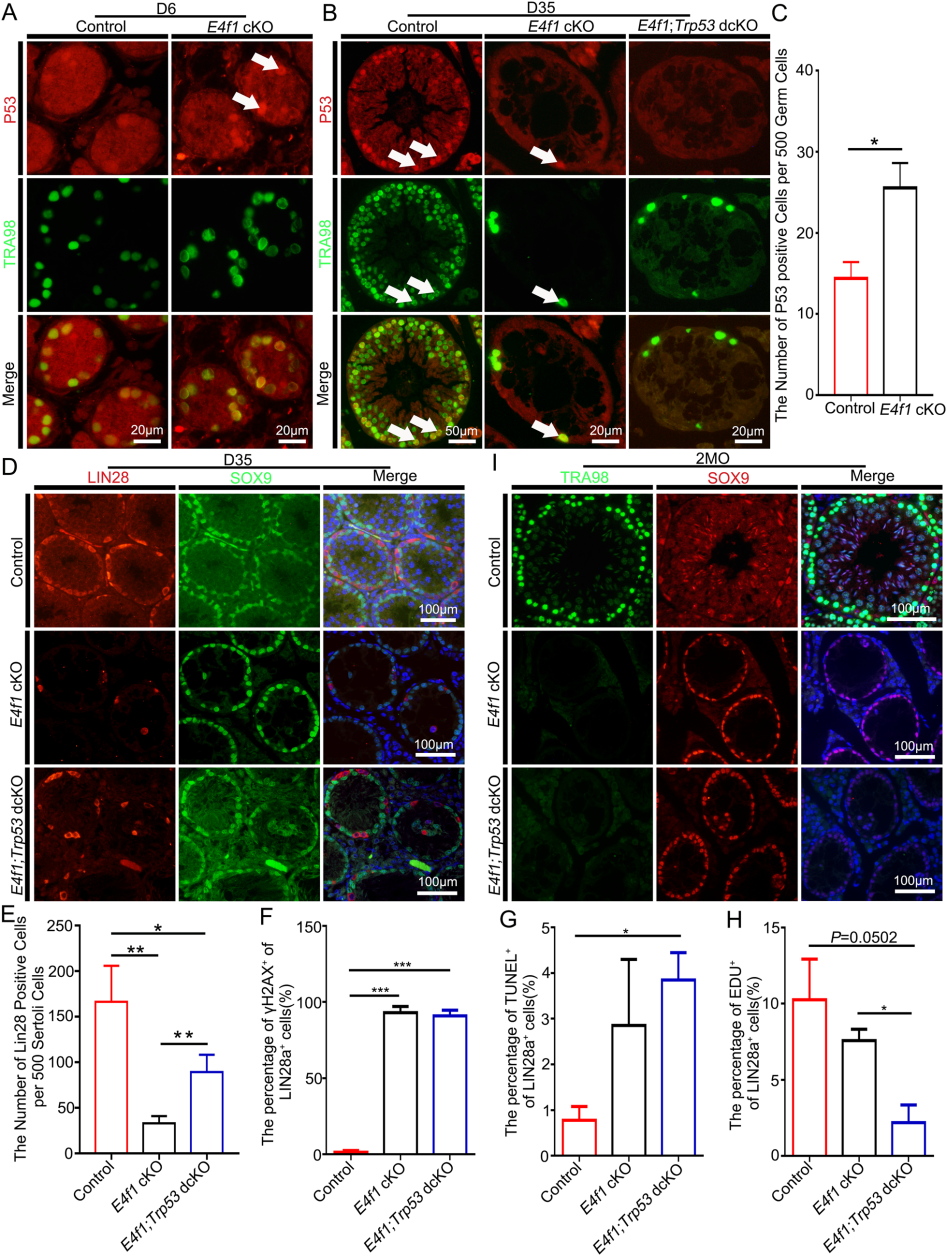
Spermatogonia loss induced by *E4f1* inactivation is not dependent on P53. **A** Immunofluorescence staining for P53(S15) (red) and TRA98 (green) in sections of PD6 controls and *E4f1* cKO testes. **B** Immunofluorescence staining for P53(S15) (red) and TRA98 in the testis of control, *E4f1* cKO and *E4f1;Trp53* dcKO testes. C Quantification of P53 (S15) per 500 germ cells in testis sections from PD6 controls and *E4f1* cKO mice. **D** Immunofluorescence staining for LIN28A (red) and SOX9 (green) in sections of PD35 control, *E4f1* cKO, and *E4f1*;*Trp53* dKO testes. **E** Quantification of undifferentiated spermatogonia per 500 Sertoli cells in testis sections from PD35 controls, *E4f1* cKO, and *E4f1*;*Trp53* dKO mice. F Percentage of γH2AX-positive undifferentiated spermatogonia in testis sections from PD35 control, *E4f1* cKO and *E4f1;P53* dcKO testes. **G** Percentage of apoptotic undifferentiated spermatogonia in testis sections from PD35 control, *E4f1* cKO and *E4f1;Trp53* dcKO testes. **H** Percentage of EdU-positive undifferentiated spermatogonia in testis sections from PD35 control, *E4f1* cKO and *E4f1;P53* dcKO testes. **I** Immunofluorescence staining for TRA98 (green) and SOX9 (red) in sections of PD60 control, E4f1 cKO, and *E4f1*;*P53* dKO testes. *P < 0.05, **P < 0.01, ***P < 0.001, Student’s t test. n = 3

Stem cell loss caused by *E4f1* inactivation in skin was rescued by *Trp53* depletion, supporting a key role of p53 in E4f1-dependent cell fate decisions [42]. *E4f1*-deficient spermatogonia manifested cell cycle arrest, DNA damage and apoptosis; therefore, we examined whether the deletion of P53 in *E4f1*-null germ cells could change the fates of SSCs and progenitors. *E4f1* inactivation did not change the total level of P53 protein; however, it significantly increased the percentage of germ cells expressing high levels of phospho-P53 (25.54 ± 3.1 vs 14.36 ± 2.03 control, n = 3, *P* < 0.05) (Fig. 9A–C), indicating the activation of P53 signaling by *E4f1* knockout [43]. We then studied the fates of spermatogonia in *E4f1;Trp53* double conditional knockout (dcKO) animals. Genotyping results showed that *E4f1* and *Trp53* were successfully deleted in *E4f1;Trp53* dcKO (Additional file 1: Fig. S5), and immunostaining confirmed that these two proteins were eliminated in germ cells (Fig. 9B). Continued studies found that the number of undifferentiated spermatogonia was increased by 1.7-fold in dcKO testes compared with *E4f1* cKO testes at PD35 (89 ± 11.14 vs 33 ± 4.58 *E4f1* cKO, n = 3, *P* < 0.01) (Fig. 9D–E). We then measured the proportion of DNA damage, proliferation and apoptosis in undifferentiated spermatogonial cells in control, *E4f1* cKO and *E4f1;Trp53* dcKO testes at PD35. Simultaneous deletion of *E4f1* and *p53* did not change the percentage of γH2AX^+^ undifferentiated spermatogonia (Fig. 9F); however, it did change the apoptosis and proliferation rates of these populations (Fig. 9G, H). Decreasing cell cycle progression likely prolonged the elimination process of undifferentiated spermatogonia because all germ cells were eventually lost in dcKO animals at PD60 (Fig. 9I), indicating that spermatogonia loss was only temporally rescued by p53 deletion. These data revealed a limited role of p53 in rescuing the developmental defects caused by *E4f1* inactivation.

## Discussion

Metabolism provides important signals to direct the self-renewal and differentiation potency of stem cells [44]. Although extensively studied in other stem cells, the relationship between metabolism and SSC fate decisions remains to be explored. It was recently proposed that SSC maintenance relies on glycolysis and that OXPHOS is important for spermatogonial differentiation [14]; however, functional experiments are required to dissect the metabolic pathways that dictate SSC homeostasis. In the present study, we described an important role of the transcription factor E4F1 in controlling SSC maintenance in mice. E4F1 directly binds to and regulates genes that encode key components of mitochondrial respiratory chains and cell cycle progression. Genetic ablation of *E4f1* in the germline impaired SSC development and fate decisions. Specifically, *E4f1* deletion led to exhaustion of the SSC pool by inducing cell cycle arrest, apoptosis, and defects in mitochondrial function. Taken together, the findings of the present study established a role for E4F1-dependent metabolism in SSC self-renewal and differentiation.

E4F1 is a multifunctional protein that has distinct roles in stem cell fate decisions. E4F1 was originally described as a potent regulator of cell cycle progression, and it physically or genetically interacts with Bmi1, pRB and p53 to control cell proliferation [45, 46]. In this study, we found that *E4f1* deficiency in spermatogonia resulted in cell cycle arrest in G1 phase and that *E4f1* likely binds to the cell cycle genes *Chek1* and *Cdk8* to fulfill its role in cell cycle progression. *Chek1* is regulated by *E4f1*, and its ectopic expression completely rescued E4F1-induced defects in hematopoietic progenitor cells [20]. In the male germline, *Chek1* deletion in germ cells caused complete depletion of all germ cells during the gonocyte to spermatogonial transition in neonatal testes [47]. This phenotype is different from that of *E4f1*cKO animals, in which undifferentiated spermatogonia were established and then gradually lost. Therefore, we speculated that E4F1 and CHEK1 control different cell cycle machineries in germ cells. It is possible that E4F1 interacts with pRB pathways in spermatogonia because *E2f1*, a crucial pRB binding patterner, was downregulated by *E4f1* deletion, and *pRB* deletion in the germline caused SSC depletion due to precocious differentiation [48, 49]. Stem cells respond to external signals in G1, and this specific phase of the cell cycle is permissive for the initiation of cell fate decisions [50]. Prolonged G1 phase may be related to the loss of self-renewal capacity by maximizing the exposure to differentiation-inducing signals [51]. However, we did not observe enhanced spermatogonial differentiation; instead, the percentage of c-Kit^+^ spermatogonia was significantly reduced in the testes of *E4f1* knockout animals. We propose that E4F1 has distinct functions in SSCs and progenitor spermatogonia.

E4F1 is a master regulator of metabolism in various stem cell types. *E4f1* deficiency in epidermal stem cells altered the expression of *Dlat*, a gene encoding the E2 subunit of the mitochondrial pyruvate dehydrogenase (PDH) complex, and redirection of the glycolytic flux toward lactate production [25]. In this study, *E4f1* deficiency reduced *Dlat* expression but only slightly changed pyruvate and lactate production. In sharp contrast, fatty acid contents were significantly increased. It is possible that fatty acid oxidation (FAO) is inhibited, therefore causing fatty acid accumulation in *E4f1*-deficient spermatogonia. FAO is essential for the maintenance of adult stem cells [52]. For example, hematopoietic stem cells (HSCs) rely on FAO for asymmetric division to maintain the quiescent stem cell compartment [53]. Neural stem cells in the resting state have a higher rate of FAO, and sustaining the quiescent state requires FAO activities [54, 55]. FAO inhibition using pharmacologic approaches increased apoptosis in spermatogonia [56], indicating a role of FAO in germ cell development; however, how *E4f1*-dependent FAO is linked to SSC fate decisions needs further investigation.

It was indicated that SSCs reside in a hypoxic niche and prefer to utilize glycolysis to sustain their regeneration capacity, while progenitors require OXPHOS for differentiation [14]. The findings of the present study do not contradict this hypothesis but provide evidence that mitochondrial function and OXPHOS are also indispensable for SSC maintenance. We found that *E4f1* inactivation directly led to significant decreases in the relative abundances of OXPHOS-related genes, such as *Ndufs5, Cox7a2, Cox6c,* and the mitochondrial inner membrane gene *Dnajc19,* in undifferentiated spermatogonia. Impairments in mitochondrial morphology and membrane potential and a reduction in ROS production further support the statement that OXPHOS was impaired. *E4f1*-deficient spermatogonia failed to transition into cKit^+^ differentiating spermatogonia, or nascent cKit ^+^ cells cannot survive without the function of *E4f1*. Nonetheless, this finding was in line with the observation that mitochondrial activities are essential for spermatogonial differentiation [16]. However, exhaustion of the SSC population indicated that OXPHOS was also required for the maintenance of stem spermatogonia. Although E4F1 is an important regulator of p53-dependent metabolic function [57], we found that deletion of p53 only temporally rescued the phenotype of *E4f1*-deficient spermatogonia and that treating *E4f1* cKO testes with a physiological concentration of H_2_O_2_ did not change the proliferation defects of these spermatogonia. Therefore, p53-mediated rescue appears independent of mitochondrial function and metabolism programs in spermatogonia. Future studies are required to elucidate the molecular pathways that are employed by p53 and E4f1 in the germline.

In summary, our study uncovered novel roles of E4F1 in regulating SSC maintenance and commitment to differentiation in mice. These findings not only provide new insights into the molecular mechanisms that link metabolism and SSC fate decisions but also shed light on cell cycle regulation in the undifferentiated spermatogonial population.

## Conclusions

In this study, we identified a list of transcription regulators that have potential roles in the fate transitions of undifferentiated spermatogonia in mice. Functional experiments demonstrated that the E4F1-mediated transcription program is a crucial regulator of metabolism and SSC fate decisions in mammals.

## Materials and methods

### Animals

All animal procedures were conducted in accordance with the Guide for the Care and Use of Laboratory Animals and were approved by the Animal Welfare and Ethics Committee at the Northwest Institute, Chinese Academy of Sciences. *Vasa-*Cre (JAX Stock No. 006954) mice were intercrossed with *E4f1*^*floxed/floxed*^* mice* (generously provided by Dr. Sauvageau from the University of Montreal) [20] to generate *Vasa*-Cre; *E4f1*^*flox/*+^ male mice. *Vasa*-Cre; *E4f1*^*flox/*+^ male mice were mated with *E4f1*^*flox/*+^ mice to generate *Vasa*-Cre; *E4f1*^*flox/flox*^ male mice (*E4f1* cKO). *Vasa*-Cre;*E4f1*^*flox/*+^ male mice were used as controls. To generate *E4f1;Trp53* conditional knockout mice, *Vasa*-Cre;*E4f1*^*flox/*+^ male mice were mated with *Trp53*^*flox/flox*^ (JAX Stock No. 008168) female mice to produce *Vasa*-Cre;*E4f1*^*flox/*+^;*Trp53*^*flox/*+^ males, which were then mated with *E4f1*^*flox/flox*^*; Trp53*^*flox/flox*^ to generate *Vasa*-Cre; *E4f1*^*flox/flox*^*; Trp53*^*flox/flox*^ (*E4f1;Trp53* dcKO). All mice were maintained on a mixed 129S2/SvPasCrl; FVB/N genetic background.

### Histological and immunohistochemistry

Histological analysis of testicular tissues was conducted as described previously [58]. Briefly, testes were fixed in Bouin’s solution for histology or 4% paraformaldehyde (PFA) for immunofluorescence staining. After dehydration, the tissues were embedded in paraffin. Paraffin-embedded tissues were then cut into 5 µm sections (Leica RM2235). After rehydration, sections were stained with hematoxylin and eosin (H&E) for histological examination under a microscope (Nikon ECLIPSE E200) or boiled in 10 mM sodium citrate (pH 6.0) for 20 min to retrieve antigen for immunofluorescence staining. The sections were washed in phosphate buffered saline (PBS) for 5 min three times and then incubated in 10% blocking serum for 1 h at room temperature. The sections were incubated with primary antibodies (Additional file 10: Table S8) overnight at 4 °C, washed in PBS for 10 min three times and incubated with secondary antibodies for 1 h at room temperature. To detect the immunofluorescent signal, DAPI was added to the slides for 1 min, and the slides were then washed in PBS. Digital images were captured with a microscope (Leica, Germany).

### Whole-mount immunofluorescence staining

Testes were stripped of the tunica albuginea and fixed for 4 h in 4% paraformaldehyde at 4 °C. Tissues were dehydrated in different grades of methanol in PBST (1% Triton X-100 in PBS) and stored in 100% methanol at − 20 °C. Tissues were rehydrated in PBST for 30 min, blocked in blocking solution (10% donkey serum and 3% BSA in PBST) for 1 h at room temperature (RT), and incubated in diluted primary antibody overnight at 4 °C. After washing with PBST, tissues were incubated in diluted secondary antibody for 2 h at RT and stained with DAPI (4′,6-diamidino-2-phenylindole) prior to visualizing the signal under a microscope (Leica, Germany).

### Proliferation and apoptosis analyses

Mice were treated with EdU (RiboBio, China) at a dosage of 50 mg/kg body weight via intraperitoneal injections. Two hours after EdU injections, testes were collected and fixed in 4% PFA. EdU incorporation was detected in cross-sections of testes using a Cell Light™ EdU Apollo 567 in vivo Kit (RiboBio, China). Apoptotic cells were detected using an In Situ Cell Death Detection Kit (Roche, Switzerland). After EdU labeling or terminal deoxynucleotidyl transferase dUTP nick end labeling (TUNEL), cross-sections were costained with Lin28 to identify proliferative or apoptotic germ cells.

### THY1^+^ spermatogonia isolation

Single-cell suspensions of testis cells were subjected to magnetic-activated cell sorting (MACS) using an antibody recognizing THY1 (Miltenyi Biotec, CD90.2 Microbeads) to isolate THY1^+^ spermatogonia. Briefly, testes from PD6 male mice were stripped of the tunica albuginea. Testes were digested with 5 ml of trypsin enzyme for 5 min at 37 °C, 1 ml of 1 mg/ml DNase I was added, and the samples were aspirated by pipette. This operation was repeated until the testes were digested into single cells. Adherent cells were abandoned by 40 μm cell strainers. The single-cell suspensions were pelleted by centrifugation at 400 × g for 5 min and resuspended in DPBS, and this operation was repeated. Then, single-cell suspensions were incubated with rat anti-mouse CD90.2 antibody (anti-THY1, 1:10 dilution; Miltenyi Biotec, Germany) in DPBS-S (1% fetal bovine serum (Gibco, USA), 10 mM HEPES (Sigma, USA), 1 mM Pyrvate (Sigma, USA), 1 mg/ml glucose (Sigma, USA), 100 u/ml penicillin (Gibco, USA), 100 u/ml streptomycin (Gibco, USA) in DPBS (Gibco, USA)) at 4 °C for 40 min. The cells were further sorted on an MS separation column (Miltenyi Biotec) according to the manufacturer’s instructions, and position cells were collected.

### PI (propidium iodide) staining and cell cycle assay

Approximately 1 × 10^5^ THY1^+^ cells were collected and fixed at 4 °C with cold ethanol for at least 2 h. The cells were then pelleted by centrifugation at 400 × *g* for 5 min and resuspended in cold PBS, and this process was repeated. Cells were resuspended in propidium iodide (PI) staining buffer (20 μg/ml PI, 0.5 mM EDTA, 0.5% NP40, 0.2 mg/ml RNase An in PBS). Cells were incubated in PI staining buffer for 2 h at 37 °C in the dark. After incubation, PI fluorescence was analyzed on a Melody flow cytometer (BD Bioscience) to determine the percentage of cells in each stage of the cell cycle with FlowJo software.

### Apoptosis assay with annexin V staining

Approximately 1 × 10^5^ THY1^+^ cells were pelleted by centrifugation at 400 × *g* for 5 min immediately after magnetic-activated cell sorting of THY1^+^ cells and incubated with Annexin V staining solution for 15 min at room temperature as described in the manufacturer’s instructions. Then, Annexin V fluorescence was analyzed on FlowSight (Ambion, USA).

### Single-cell ATAC-Seq

Lin28^+^ spermatogonia were isolated for scATAC-seq. The isolation, washing, and counting of nuclei suspensions were performed according to the Demonstrated Protocol: Nuclei Isolation for Single Cell ATAC Sequencing (10 × Genomics; CG000169). Nuclei were then immediately used to generate 10 × single-cell ATAC libraries. The nuclei suspension was loaded into the Chromium microfluidic chip E with 10 × reagents and barcoded with a 10 × Chromium Controller (10 × Genomics, Pleasanton, CA). DNA fragments from the barcoded cells were subsequently amplified, and sequencing libraries were constructed with reagents from a Chromium Single Cell ATAC reagent kit (10 × Genomics; PN-1000110, PN-1000156, PN-1000084) according to the manufacturer’s instructions. Libraries were then pooled and loaded on the Illumina 2000 platform to produce raw data. Then, the raw data were analyzed by Cell Ranger ATAC (10 × Genomics, version 1.1.0, https://support.10xgenomics.com/single-cell-atac/software/pipelines/latest/algorithms/overview) using default parameters. Raw sequencing data were aligned to the reference genome, read filtering and alignment were performed, and accessible chromatin peaks were detected. Finally, raw data produced a filtered peak-by-cell matrix by the Cell Ranger ATAC pipeline.

The filtered peak-by-cell matrix was imported into ArchR [59] (https://github.com/GreenleafLab/ArchR). A quality control cutoff of a minimum of 1000 fragments per cell was used in the peak region. We conducted filtering doublets with “LSI” knnMethod. Next, ArchR performed dimensionality reduction with latent semantic indexing (LSI), and the first 30 components were calculated. Then, the graph clustering method implemented by Seurat was used for the identification of clusters with “nNeighbors = 30, minDist = 0.5 and metric = cosine”. Then, uniform manifold approximation embedding (UMAP) was used to visualize single cells in reduced dimension space. We estimated gene expression (gene score) for these genes from our chromatin accessibility data by the addGeneScoreMat function. We identified marker peaks with “cutoff = “FDR <  = 0.01&Log2FC >  = 0.5””. We looked for motifs that are enriched in peaks in various cell types using the CIS-BP Dataset, and “cutoff = “FDR <  = 0.1&Log2FC >  = 0.5””. For integrative analysis of scRNA-seq and scATAC-seq, we compared the scATAC-seq gene score matrix with the scRNA-seq gene expression matrix with ArchR’s addGeneIntegrationMatrix function. We retrieved peak-to-gene links by using the getPeak2GeneLinks function with “corCutOff = 0.45” and “resolution = 1” and identified TFs whose motif accessibility was correlated with their own gene activity (by gene score) by using the correlateMatrices function.

### Single-cell RNA-seq

Testes from control and *E4f1* cKO male mice at PD6 were digested with 5 mL tyrisin enzyme for 5 min at 37 °C, 1 ml 1 mg/ml DNase I was added, the samples were blown by pipette, and this operation was repeated until the testes were digested into single cells. Adherent cells were removed with 40 µm cell strainers. The single-cell suspension was pelleted by centrifugation at 400 × *g* for 5 min and resuspended in 5 ml of DPBS-S. A 5 ml cell suspension was added to 2 ml of 30% Percoll (Sigma, USA) in DPBS-S and centrifuged at 1800 rpm for 8 min to remove Sertoli cells and interstitial cells. Cells at the bottom of the centrifuge tube were collected, and red blood cells were removed using Red Blood Cell Lysis Buffer (Solarbio, China). The remaining cells were used for single-cell RNA-seq.

We performed single-cell capture and cDNA synthesis with the BD Rhapsody™ Single-Cell Analysis System (Doc ID: 210966 Rev.1.0 PROTOCOL) to capture single cells and synthesize cDNA. Library preparation was carried out according to BD Rhapsody™ System mRNA Whole Transcriptome Analysis (WTA) and AbSeq Library Preparation Protocol. Paired-end sequencing (150 bp) was performed on the Illumina HiSeq 2000 platform (sequenced by Novogene).

Quality control and analysis of raw data were conducted according to the BD Rhapsody pipeline. Pipeline filtered and removed low-quality reads that met the following criteria: read1 length < 66 bp or read2 length < 64 bp; mean base quality score < 20 for read1/read2; read 1 of highest single nucleotide frequency (SNF) ≥ 0.55 or read2 of SNF ≥ 0.80. Read 1 included a cell label including 3 sections and 2 linker sequences, a unique molecular index (UMI) including 8 random bases, and a poly T tail. A read1 had to have 3 components for it to be retained: perfect match to predefined cell label sequence at expected locations; UMI with non-N bases; and at least 6 T in the 8 bases following UMI. Read 2 was aligned to the mm 10 mouse transcriptome (UCSC) using Bowtie 2. A read2 kept has the following criteria: the read aligns uniquely to a gene sequence in the reference; the alignment begins within the first 5 bases; the length of the alignment that can be a match or mismatch in the CIGAR string is > 60; and the read does not align to phiX174. Then, the pipeline combined information from read 1 and read 2. Reads with the same cell label, same UMI sequence, and same gene were collapsed into a single raw molecule. To reduce the impact of PCR and sequencing errors on the number of raw molecules, the BD Phapsody pipeline used recursive substitution error correction (RSEC) to correct errors caused by base substitutions and distribution-based error correction (DBEC) to adjust for errors derived from library preparation steps or sequencing base deletions. The BD pipeline identifies the number of cells based on the second-order import algorithm.

The cell-gene expression matrix file produced by the BD Phapsody pipeline was imported into R, and we used the Seurat package to conduct quality control, cell selection, data normalization, variable gene analysis, PCA, dimensional reduction, clustering of the cells, DEG identification, and functional analysis of DEGs. The Monocle 2 package was used to analyze pseudotime, and the scran package was used to analyze the cell cycle.

### Metabolites related to TCA and fatty acid assay

Approximately 1 × 10^5^ THY1^+^ cells were used for metabolite assays by GC‒MS. One milliliter of methanol (Merck, Germany)/H_2_O (4:1, v/v, containing 10 μg/mL tridecanoic acid as an internal standard) was added to a 1.5 ml centrifuge tube. After vortexing and centrifugation, the supernatant of each sample was lyophilized. After derivatization with methoxyamine pyridine followed by N-methyl-N-(trimethylsilyl)-trifluoroacetamide (MSTFA) (Sigma, USA) at 37 °C for 1 h in a water bath, the samples were centrifuged, and the supernatants were used for GC‒MS SIM analysis. A QP 2010 GC‒MS plus system with an AOC-20i automatic injector (Shimadzu, Japan) coupled with a DB-5 MS fused-silica capillary column (30 m × 0.25 mm × 0.25 μm, Agilent Technologies, USA) was used for GC‒MS analysis. The column temperature was held at 70 °C for 3 min, increased to 300 °C at a rate of 5 °C/min, and then held for 10 min. The injection temperature, transfer line and ion source were maintained at 300 °C, 280 °C and 230 °C, respectively. One microliter of 1 µl of sample was injected at a split ratio of 1:10. The carrier gas, helium (99.9995%, China), was maintained at a constant linear velocity of 40 cm/s, and the electron ionization source voltage was 70 eV. Data acquisition started at 5.0 min.

### Mitochondrial ROS detection assay

THY1^+^ cells isolated by MACS were immediately pelleted by centrifugation at 400 × g for 5 min and resuspended in 37 °C HBSS containing calcium and magnesium (14025076, Thermo Fisher Scientific, USA). MitoSOX™ Red (50 μg, M36008, Thermo Fisher Scientific, USA) diluted with 13 μl dimethyl sulfoxide (DMSO) was added to 1 μl of the 1 mL cell suspension and incubated for 30 min at 37 °C, and the fluorescence intensity of MitoSOX™ Red staining was measured by flow cytometry (FlowSight, Merck Millipore, Germany).

### TEM of testes

Testes from PD 6 male mice were cut into two pieces and fixed in 2.5% glutaraldehyde. Then, testis pieces were postfixed in 1% OsO_4_ for 2 h. After rehydration, testis pieces were embedded in epoxy resin. Testis pieces were cut into 80 nm ultrathin sections, and the sections were collected on formvar-coated copper grids. These grids were counterstained with uranyl acetate and lead citrate and examined on an EM2010FEF-Ω transmission electron microscope (JEOL, Tokyo, Japan).

### Mitochondrial membrane potential (MMP) measurement

JC-1 is a cationic dye that accumulates in mitochondria. This accumulation is dependent on the membrane potential and indicated by a fluorescence emission shift from green (525 nm) to red (529 nm). Mitochondral membrane potential was indicated by the red/green fluorescence intensity ratio. After 20 min of incubation with JC-1 (C2006, Beyotime, China) at 37 °C, THY1^+^ cells were washed twice with PBS. The fluorescence intensity of THY1^+^ cells was detected using FACS (FlowSight, Merck Millipore, Germany).

### ChIP‒qPCR

Testicular cells (1 × 10^7^ ~ 2 × 10^7^) from 10 to 20 PD6-PD8 mouse litters were filtered with 30% Percoll, cross-linked with 1% formaldehyde for 10 min at room temperature and quenched by the addition of 125 mM glycine for 15 min. The cells were scraped, pelleted, and washed with wash buffer (20 mM Na-butyrate and 1 × proteinase inhibitor cocktail in PBS). Cell pellets were suspended in 1000 µl lysis buffer (10 mM Tris, 100 mM NaCl, 1 mM EDTA, 0.5 mM EGTA, 0.1% Na-deoxycholate, 0.5% N-lauroylsarcosine in ddH2O) and placed on a rocking platform for 10 min at 4 °C. Chromatin was sheared via sonication (avg 300–500 bp). The sonicated sample was centrifuged to pellet the cellular debris. The supernatant was transferred to three fresh 1.5 mL tubes, with 300 μL per tube. Thirty microliters of supernatant was stored in a − 20 °C refrigerator as input. Aliquots of chromatin were incubated with 5 μg of antibodies against E4f1 (mouse anti-E4f1, sc-11146, Santa Cruz Biotechnology), RNA polymerase II phospho-CTD-Ser2 (mouse anti-POLII-pSer2-CTD, 61083, Active Motif, USA) or mouse normal IgG at 4 °C overnight. A 50 μL aliquot of Protein G/magnetic beads was washed three times with cold blocking solution (0.5% BSA in PBS) and used to isolate immune complexes from E4f1, RNA polymerase II and IgG IPs. Then, the chromatin-bound beads were washed four times in 1 mL of cold RIPA wash buffer (50 mM HEPES, 500 mM LiCl, 1 mM EDTA, 1% NP-40, 0.7% Na-deoxycholate in ddH2O), 1 mL of TE/50 mM NaCl was added to the tube, and the chromatin-bound beads in TE/50 mM NaCl were transferred to a fresh 1.5-mL microfuge tube. All TEs were removed, eluted with 210 μL elution buffer (50 mM Tris, 10 mM EDTA, 1% SDS in ddH_2_O) for 15 min at 65 °C, and treated with both RNase A and proteinase K. Following overnight incubation at 65 °C to reverse crosslinks, ChIP DNA was purified by phenol‒chloroform extraction and ethanol precipitation. Immunoprecipitated DNAs were analyzed by qPCR (ABI ViiA7, SYBR Green) with promoter-specific primers for the E4f1-bound regions, and ChIP qPCR was used to analyze the enrichment of E4f1. RNA polymerase II-bound genomic regions of *Gapdh* (C17021045-500, DAKEWE, China) were used as positive controls (Additional file 7: Table S5).

### Statistics

Assessment of statistical significance was performed using a two-tailed unpaired t test for normally distributed data and two-tailed Mann‒Whitney U tests or Kruskal‒Wallis with Dunn’s multiple comparisons tests for data that were not normally distributed. All statistical analyses were performed using GraphPad Prism v7. Statistical significance is expressed as follows: *P < 0.05, **P < 0.01, ***P < 0.001.

### Supplementary Information

**Additional file 1: Figure S1** Expression and function of E4F1 during prespermatogonia to spermatogonial transition. (A) Immumohistochemical staining for E4F1 in sections of testes from PD0 and PD6 mice. n=4. The black arrows indicate E4F1 negative cells and the red arrows indicate E4F1 positive cells. (B) The Percentage of E4F1^+^ Cells in Germ Cells in Figure (A). (C) The expression of *E4f1* mRNA from controls and *E4f1* cKO testes. (D) Immunofluorescence staining for TRA98 (green) in sections of 2MO *E4f1* cKO testes(panorama). n=2. (E) Immunofluorescence staining for GFRA1 (red) and TRA98 (green) in sections of PD6-8 control and *E4f1* cKO testes. n=4. (F) Quantification of progenitor spermatogonia per 500 germ cells in sections of PD6-8 control and *E4f1* cKO testes. n=4. (G) Immunofluorescence staining for TRA98 (green) in sections of PD3 and PD6 control and *E4f1* cKO testes. n=4. (H) Percentage of germ cells located in basement membrane of PD3 (left) and PD6 (right) control and *E4f1* cKO testes. n=4. **Figure S2** Impact of *E4f1* deletion on cell cycle progression and apoptosis. (A) Flow cytometry analysis of cell cycle in THY1^+^ cells from controls and *E4f1* cKO testes by PI staining. (B) Histogram of cell cycle distribution of THY1+ cells from controls and *E4f1* cKO testes. n=3. (C) Flow cytometry analysis of apoptotic cells in THY1^+^ cells from controls and *E4f1* cKO testes by Annexin V staining. (D) Percentage of Annexin V positive cells in THY1^+^ cells from controls and E4f1 cKO testes. n=2. Error bars represent SD. *p < 0.05, Student t test. **Figure S3** Quality control of scRNA-seq data. (A)UMAP plot of cells from D6 control mouse testes and representative markers for each cell type. (B) UMAP plot of cells from D6 *E4f1* cKO mouse testes and representative markers for each cell type. (C) Distribution of basic cell information of each sample before filtering, including the number of detected genes (Y-axis) in each sample; Distribution of the total number of UMI detected in a single cell of each sample (Y-axis);Percentage (Y-axis) distribution of mitochondrial gene expression in individual cells of each sample. **Figure S4** Supplementation of H_2_O_2_ did not rescue proliferative defects of *E4f1*-deficient germ cells. (A) Images of MitoSOXTM red staining for THY1^+^ cells from controls and *E4f1* cKO testes by fluorescence microscope (up) and FACS analysis (down). (B) Immunofluorescence staining of LIN28A(green) and EdU(red) in control and *E4f1* cKO testicular sections supplemented with H_2_O_2_ and cultured for 2 days. -H_2_O_2_ indicates control and+H_2_O_2_ means cultured tissued were treated with H_2_O_2_.n=3. (C) Percentage of EdU^+^ spermatogonia in control and *E4f1* cKO testicular sections supplemented with H2O2 and cultured for 2 days. n=3. **Figure S5** Genotyping results of *Trp53*; *E4f1* double knockout animals (A) Images of genotypic identification.Trp53: flox/flox 584bp; WT 431bp. E4f1: flox/flox 208bp; WT 140bp. Vasa-cre: cre 240bp; WT 324bp.

**Additional file 2. Supplemental material: testis tissue culture**

**Additional file 3: Table S1**. scATAC-seq peaks in different clusters of Lin28^+^ spermatogonia.

**Additional file 4: Table S2**. Gene Score of scATAC-seq in different Lin28^+^ spermatogonia clusters.

**Additional file 5: Table S3**. Prediction of Transcription regulators based on GeneScores.

**Additional file 6: Table S4**. Differentially expressed genes in 9 different states.

**Additional file 7: Table S5**. Differentially expressed genes in SSCs, progenitors and differentiating spermatogonia. 

**Additional file 8: Table S6**. KEGG analysis of differentially expressed genes between control and E4f1 cKO germ cells. 

**Additional file 9: Table S7**. Predicted E4F1 target genes.

**Additional file 10: Table S8**. Information of antibodies and primers used in this study.

## Data Availability

The authors declare that the data supporting the findings of this study are available within the paper and its Additional file 2 or are available from the corresponding author upon reasonable request. Single-cell RNA-seq data have been uploaded to NCBI (SRA: SUB11619849, BioProject: PRJNA849719).
