# Supplementary material for: Transcription factor E4F1 dictates spermatogonial stem cell fate decisions by regulating mitochondrial functions and cell cycle progression
Source: Cell Biosci. 2023 Sep 25;13:177. doi: 10.1186/s13578-023-01134-z (PMC10521505; doi:10.1186/s13578-023-01134-z)
Supplement: Supplementary file 1 — Additional file 1: Figure S1 Expression and function of E4F1 during prespermatogonia to spermatogonial transition. (A) Immumohistochemical staining for E4F1 in sections of testes from PD0 and PD6 mice. n=4. The black arrows indicate E4F1 negative cells and the red arrows indicate E4F1 positive cells. (B) The Percentage of E4F1+ Cells in Germ Cells in Figure (A). (C) The expression of E4f1 mRNA from controls and E4f1 cKO testes. (D) Immunofluorescence staining for TRA98 (green) in sections of 2MO E4f1 cKO testes(panorama). n=2. (E) Immunofluorescence staining for GFRA1 (red) and TRA98 (green) in sections of PD6-8 control and E4f1 cKO testes. n=4. (F) Quantification of progenitor spermatogonia per 500 germ cells in sections of PD6-8 control and E4f1 cKO testes. n=4. (G) Immunofluorescence staining for TRA98 (green) in sections of PD3 and PD6 control and E4f1 cKO testes. n=4. (H) Percentage of germ cells located in basement membrane of PD3 (left) and PD6 (right) control and E4f1 cKO testes. n=4. Figure S2 Impact of E4f1 deletion on cell cycle progression and apoptosis. (A) Flow cytometry analysis of cell cycle in THY1+ cells from controls and E4f1 cKO testes by PI staining. (B) Histogram of cell cycle distribution of THY1+ cells from controls and E4f1 cKO testes. n=3. (C) Flow cytometry analysis of apoptotic cells in THY1+ cells from controls and E4f1 cKO testes by Annexin V staining. (D) Percentage of Annexin V positive cells in THY1+ cells from controls and E4f1 cKO testes. n=2. Error bars represent SD. *p < 0.05, Student t test. Figure S3 Quality control of scRNA-seq data. (A)UMAP plot of cells from D6 control mouse testes and representative markers for each cell type. (B) UMAP plot of cells from D6 E4f1 cKO mouse testes and representative markers for each cell type. (C) Distribution of basic cell information of each sample before filtering, including the number of detected genes (Y-axis) in each sample; Distribution of the total number of UMI detected in a single c [file 13578_2023_1134_MOESM1_ESM.docx]

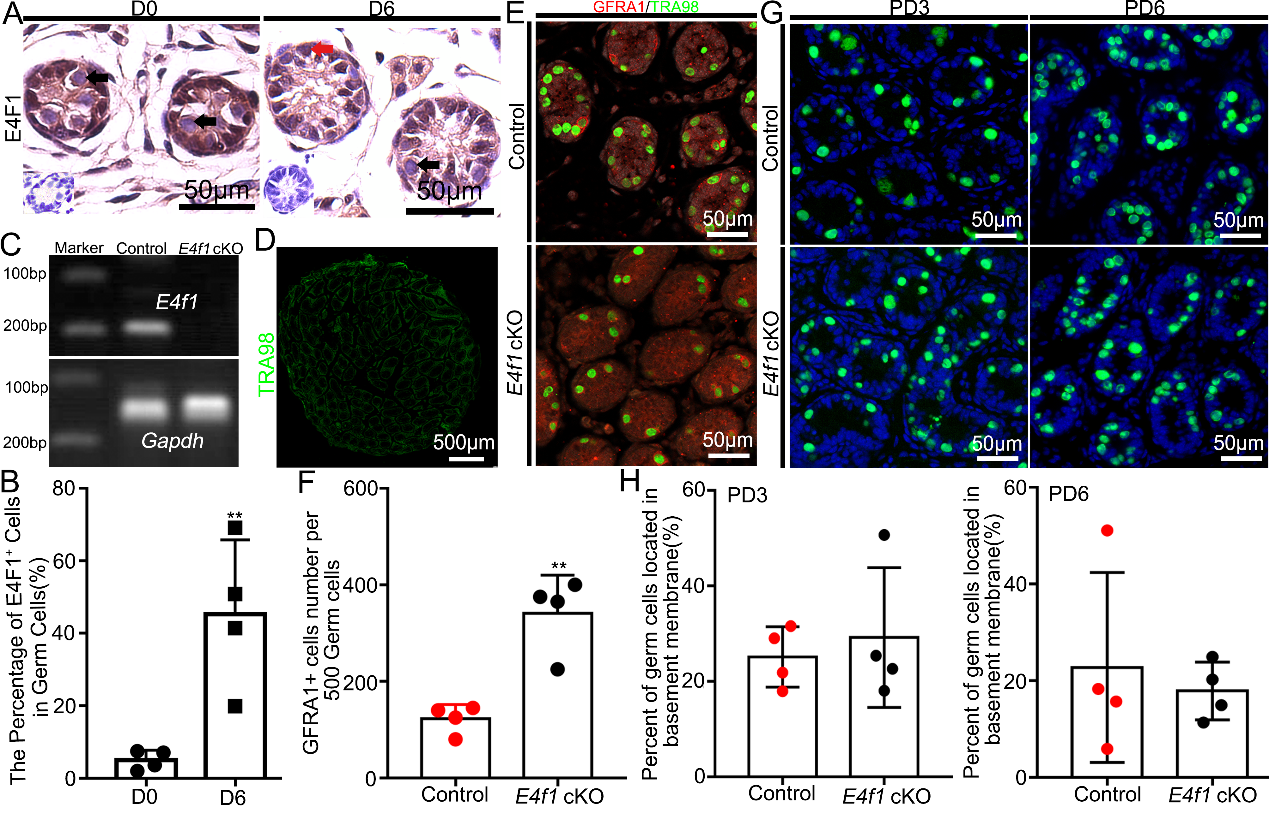
**Supplementary Figure S1** (**A**) Immumohistochemical staining for E4F1 in sections of testes from PD0 and PD6 mice. n=4. The black arrows indicate E4F1 negative cells and the red arrows indicate E4F1 positive cells. (**B**) The Percentage of E4F1^+^ Cells in Germ Cells in Figure (A). **(C)** The expression of *E4f1* mRNA from controls and *E4f1* cKO testes. **(D)** Immunofluorescence staining for TRA98 (green) in sections of 2MO *E4f1* cKO testes(panorama). n=2**. (E)** Immunofluorescence staining for GFRA1 (red) and TRA98 (green) in sections of PD6-8 control and *E4f1* cKO testes. n=4. **(F)** Quantification of progenitor spermatogonia per 500 germ cells in sections of PD6-8 control and *E4f1* cKO testes. n=4. **(G)** Immunofluorescence staining for TRA98 (green) in sections of PD3 and PD6 control and *E4f1* cKO testes. n=4. **(H)** Percentage of germ cells located in basement membrane of PD3 (left) and PD6 (right) control and *E4f1* cKO testes. n=4.


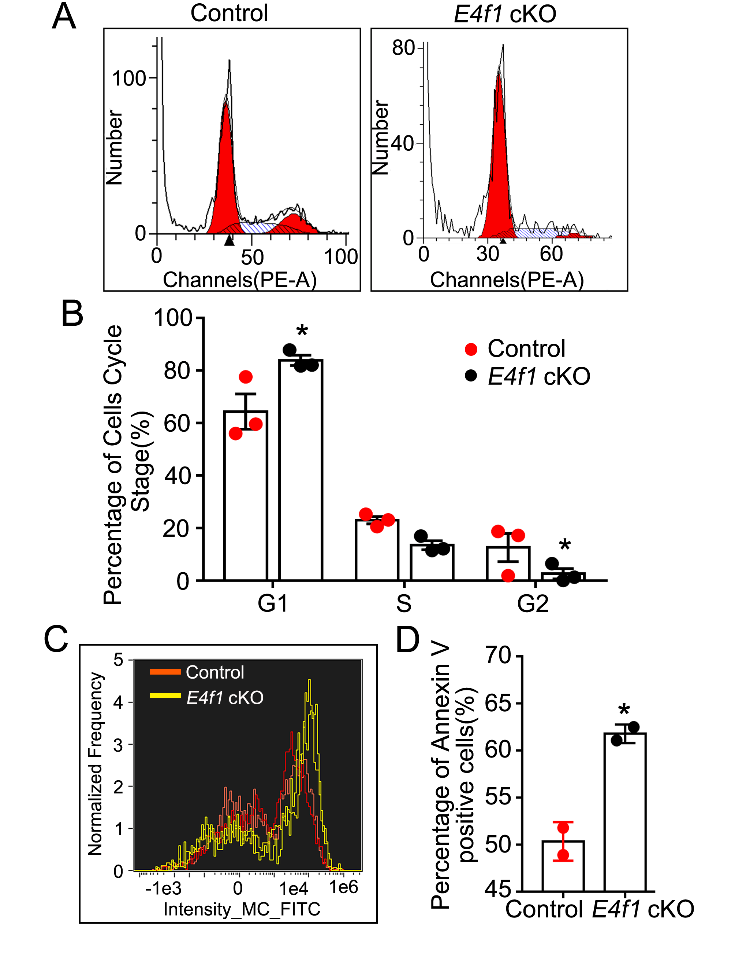
**Supplementary Figure S2** (**A**) Flow cytometry analysis of cell cycle in THY1^+^ cells from controls and *E4f1* cKO testes by PI staining. (**B**) Histogram of cell cycle distribution of THY1^+^ cells from controls and *E4f1* cKO testes. n=3. **(C)** Flow cytometry analysis of cell apoptotic cells in THY1 positive cells from controls and *E4f1* cKO testes by Annexin V staining. (**D**) Percentage of Annexin V positive cells in THY1^+^ cells from controls and *E4f1* cKO testes. n=2. Error bars represent SD. **p* < 0.05, Student *t* test.


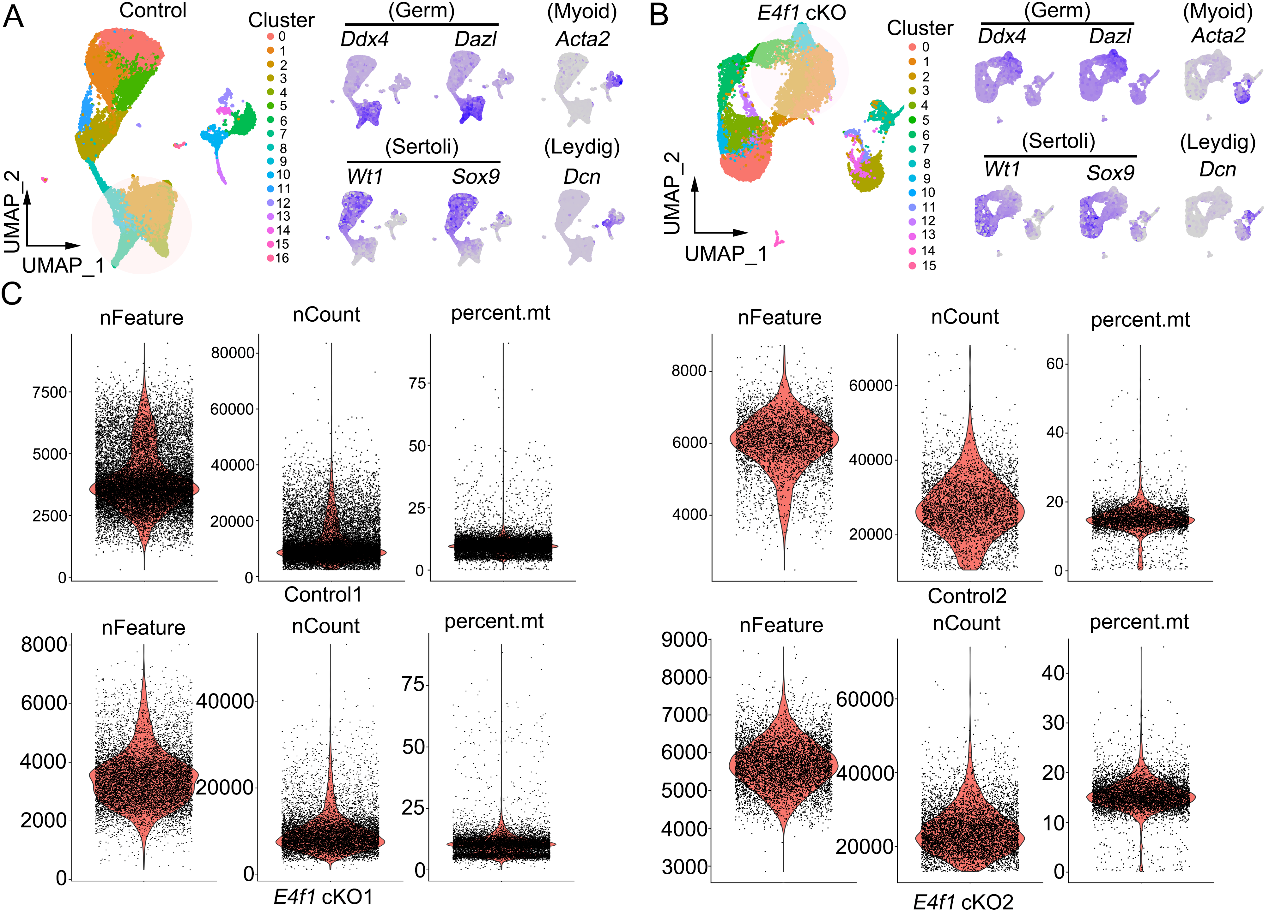
**Supplementary Figure S3** (**A**)UMAP plot of cells from D6 control mouse testes and representative markers for each cell type. **(B)** UMAP plot of cells from D6 *E4f1* cKO mouse testes and representative markers for each cell type. (**C**) Distribution of basic cell information of each sample before filtering, including the number of detected genes (Y-axis) in each sample; Distribution of the total number of UMI detected in a single cell of each sample (Y-axis);Percentage (Y-axis) distribution of mitochondrial gene expression in individual cells of each sample.


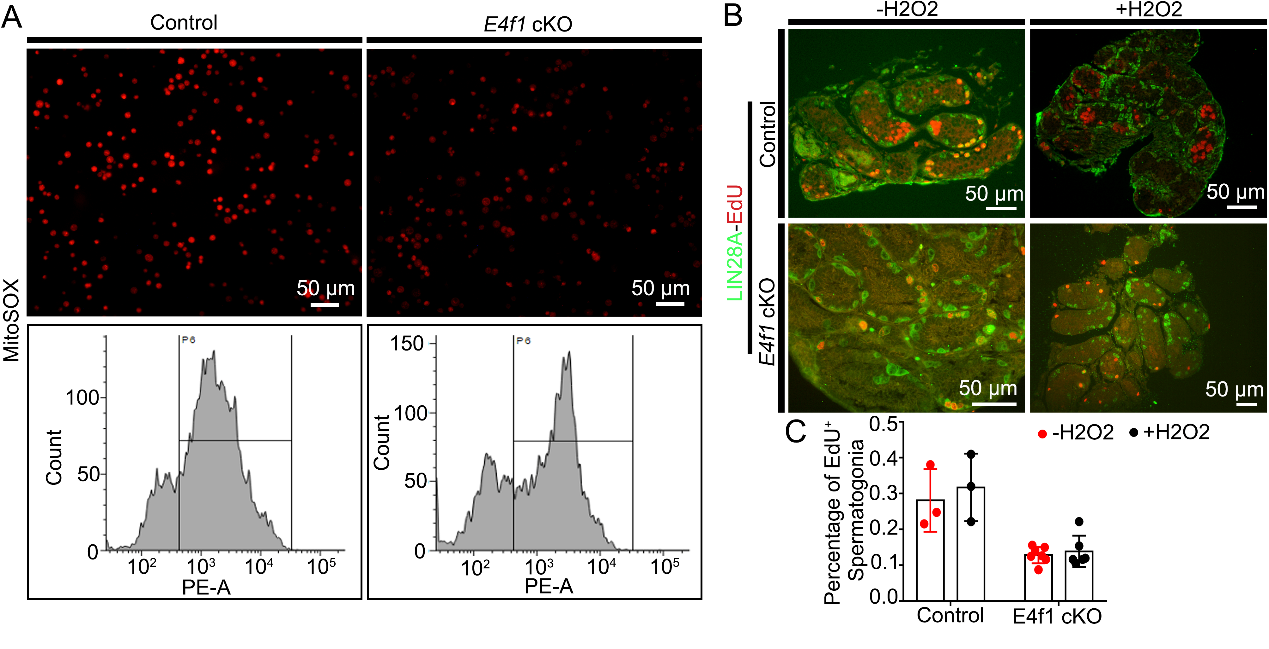
**Supplementary Figure S4** **(A)** Images of MitoSOX^TM^ red staining for THY1^+^ cells from controls and *E4f1* cKO testes by fluorescence microscope (up) and FACS analysis (down). **(B)** Immunofluorescence staining of LIN28A(green) and EdU(red) in control and *E4f1* cKO testicular sections supplemented with H2O2 and cultured for 2 days. -H2O2 indicates that H2O2 is not added. +H2O2 means add H2O2.n=3. **(C)** Percentage of EdU+ spermatogonia in control and *E4f1* cKO testicular sections supplemented with H2O2 and cultured for 2 days. n=3.

**Supplementary Figure S5** **(A)** Images of genotypic identification
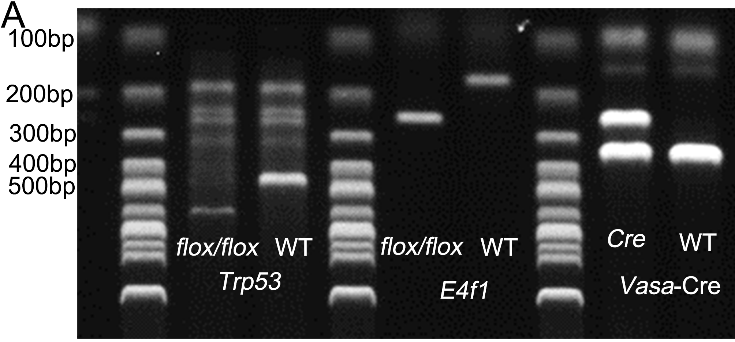
.*Trp53*: flox/flox 584bp; WT 431bp. *E4f1*: flox/flox 208bp; WT 140bp. *Vasa*-cre: cre 240bp; WT 324bp.
