# Supplementary material for: Transcription factor E4F1 dictates spermatogonial stem cell fate decisions by regulating mitochondrial functions and cell cycle progression
Source: Cell Biosci. 2023 Sep 25;13:177. doi: 10.1186/s13578-023-01134-z (PMC10521505; doi:10.1186/s13578-023-01134-z)
Supplement: Supplementary file 2 — Additional file 2. Supplemental material: testis tissue culture [file 13578_2023_1134_MOESM2_ESM.docx]

**Testis culture**

A 5 ml aqueous sterile solution of 1.5% (*w/v*) agarose was solidified into a well of a 6-well cell culture plate. Once solidified, the gel was cut into 3 hexahedron shapes of approximately 10×10×7 mm^3^ in size with a sterile blade, and the remaining gel was discarded. Three milliliters of testis culture medium was added to the preparation of 49.5 ml of Dulbecco’s Modified Eagle Medium Nutrient Mixture F-12 (DMEM/F12) (Gibco, USA), 0.5 ml of penicillin‒streptomycin (10000 u/ml penicillin; 10000 u/ml streptomycin) (Gibco, USA) and 30 μM H_2_O_2_ or not. After 2 hours, testes from PD 6 mice were cut into 1 mm^3^ pieces. The testes pieces were placed on the gel hexahedron and cultured in a 5% CO_2_, 95% air atmosphere at 37°C for 48 hours. The culture medium was changed every 24 hours. Two hours before testis piece collection, 10 μl EdU was added to the culture medium.
