# Supplementary material for: Transcription factor E4F1 dictates spermatogonial stem cell fate decisions by regulating mitochondrial functions and cell cycle progression
Source: Cell Biosci. 2023 Sep 25;13:177. doi: 10.1186/s13578-023-01134-z (PMC10521505; doi:10.1186/s13578-023-01134-z)
Supplement: Supplementary file 10 — Additional file 10: Table S8. Information of antibodies and primers used in this study. [file 13578_2023_1134_MOESM10_ESM.docx]

**Antibodies used in this study.**

| **Antibody** | **Dilution ratio** | **Company** | **Catalogue No.** |
| --- | --- | --- | --- |
| Mouse anti-E4F1 | 1:100 | Santa Cruze | Sc-514718 |
| Goat anti-LIN28A | 1:200 | R&D Systems | AF3767 |
| Rat anti-TRA98  Rabbit anti-SOX9  Goat anti-Kit  Rabbit anti-Ki67  Mouse anti-p-Histone H3  Mouse anti-H2AX | 1:200  1:200  1:200  1:200  1:200  1:400 | Abcam  Miilipore  R&D System  Abcam  Santa cruze  Abcam | ab82527  AB5535  AF1356  Ab15580  Sc-374669  5438s |
| Rabbit anti-p-P53(S15)  Normal goat IgG  Normal rabbit IgG  Normal rat IgG  Normal mouse IgG | 1:100  1:1000  1:1000  1:1000  1:1000 | Cell Signaling Technology  Sino Biological Inc  Cell Signaling Technology  Santa Cruze  Santa Cruze | 9284T  CR2  2729s  sc-2026  sc-2025 |

**Primers(5’-3’) used in this study.**

| **Genotype** |  | |  |  |
| --- | --- | --- | --- | --- |
| *E4f1* | A | | ACCTGCAGTTTGTTGTGATTG | |
|  | B  C | | CCTTGAGGCGCCACCAGG  ATGTGGCCGCTGCCAAGTG | |
| *P53* | A | | AAGGGGTATGAGGGACAAGG | |
|  | B | | GAAGACAGAAAAGGGGAGGG | |
| *Vasa*-Cre | A  B  C  D | | CACGTGCAGCCGTTTAAGCCGCGT  TGCCCATTCTAAACAACACCCTGAA  CTAGGCCACAGAATTGAAAGATCT  GTAGGTGGAAATTCTAGCATCATCC | |
| **ChIP PCR** | |  |  |  |
| *Chek1* | Forward | | AGCTGCCACAGGAATCCAAA | |
|  | Reverse | | CGGTCTACTGAAGTCGCTCC | |
| *Uqcrq* | Forward | | AGGTCAGAAGACGAGCTGTAG | |
|  | Reverse | | CAAGATAGCAACTTGCCGCC | |
| *Ndufs5*  *Ndufc1*  *Cox7a2l*  *Dlat*  *Chek2*  *Dnajc19* | Forward  Reverse  Forward  Reverse  Forward  Reverse  Forward  Reverse  Forward  Reverse  Forward  Reverse | | GGAGGACGCAGACGCC  CCGCTGTGCTTGGTTTCTAC  TCACATTGATTCCTCTCAGTCCT  GGGGCTATGTAGCTCAGTGG  CCCACAACCCGAGAATTGGT  TGTACTCTTGAGCCTGGTGC  ACAGACGCGCCACATTACTGC  GCTGCTCTTGGAGAGGTCACT  ACGCGGTGAGTCATAAGTGGGAAA  GCACAAGAAAGCAGACAAAGCGGA  AAAGTGCGTCGGAACTGTCGCACAAA  TTTAGGGCCGGATATTGGACTTGC | |

| **PCR** | |  |  |  |
| --- | --- | --- | --- | --- |
| *E4f1* | Forward | | CGGAGCATGGCACACTCAA | |
|  | Reverse | | GTCATCTGCAGTGGCCTCAA | |
